# Approaching onchocerciasis elimination in Equatorial Guinea: Near zero transmission and public health implication

**DOI:** 10.1186/s40249-024-01254-9

**Published:** 2024-11-14

**Authors:** Policarpo Ncogo, Ana Hernández-González, Thuy-Huong Ta-Tang, Lidia Redondo, Ana Álvarez, Maria J. Perteguer, José M. Rubio, Rufino Nguema, Justino Nguema, Marta García, Laura Reguero, Teresa Valverde, Marta Lanza, Laura Cerrada-Gálvez, Maria Rebollo, Jorge Cano, Agustín Benito, Zaida Herrador

**Affiliations:** 1 National Onchocerciasis and Other Filariasis Control Programme. Ministry of Health, Malabo, Equatorial Guinea; 2 National Centre for Tropical Medicine, Institute of Health Carlos III (ISCIII in Spanish), Madrid, Spain; 3 Ministry of Health, Fundación Estatal, Infancia y Bienestar Social, F.S.P. (CSAI), Madrid, Spain; 4 National Centre of Microbiology, Institute of Health Carlos III, Madrid, Spain; 5 CIBERINFEC.Institute of Health Carlos III, Madrid, Spain; 6 Global Program for Onchocerciasis Elimination & Scabies Control, Department of Control of Neglected Tropical Diseases, World Health Organization, Geneva, Switzerland; 7 Expanded Special Project for Elimination of NTDs, World Health Organization Regional Office for Africa, Brazzaville, Republic of Congo; 8 National Centre of Epidemiology, Health Institute Carlos III, Madrid, Spain; 9 CIBERESP, Institute of Health Carlos III, Madrid, Spain

**Keywords:** Onchocerciasis, Lymphatic filariasis, Mapping, PCR, Serology, Equatorial Guinea

## Abstract

**Background:**

Onchocerciasis and lymphatic filariasis (LF) are endemic in Equatorial Guinea with notable variations in disease incidence between island and mainland regions. Historically, efforts to control and map these diseases were concentrated in Bioko Island, where loiasis is absent, allowing for targeted onchocerciasis interruption strategies. With the cessation of onchocerciasis transmission on Bioko and no reported cases on Annobon island, assessing the transmission status in the previously unaddressed mainland region has become imperative. Mapping efforts in mainland Equatorial Guinea have proven low to moderate level of transmission for LF and onchocerciasis, although the results so far have not been very conclusive. The current study aims to update the prevalence estimates for onchocerciasis and LF in mainland Equatorial Guinea using various diagnostic techniques.

**Methods:**

This is the first cross-sectional study carried out to estimate the prevalence of onchocerciasis and LF in the mainland area of Equatorial Guinea, from September to December 2019, based on the combination of skin snip biopsies, thick blood smears, laboratory serological tests (ELISA tests for the detection of IgG4 antibodies against *Onchocerca volvulus* recombinant antigen Ov16 and *Wuchereria bancrofti* recombinant antigen Wb123) and molecular laboratory tests. Frequencies and prevalence rates, along with 95% confidence intervals for interval estimation of a binomial proportion, were computed.

**Results:**

The overall onchocerciasis seroprevalence calculated for the study was 0.3% (95% *CI*: 0.1 to 0.5%). Microscopic examination of skin biopsies from the eight individuals seropositive for Ov16, out of the 3951 individuals initially tested, revealed no *O. volvulus* microfilariae. However, DNA extracted from one skin snip was successfully amplified, with subsequent sequencing confirming the presence of *O. volvulus*. Among the 3951 individuals, 182 were found to have anti-Wb123 antibodies, suggesting exposure to *W. bancrofti*, with an estimated seroprevalence of 4.6% (95% *CI*: 4.0 to 5.3%). Microscopy and Filaria-real time-PCR (F-RT-PCR) analysis for *W. bancrofti* were negative across all samples.

**Conclusions:**

The findings indicate that onchocerciasis may no longer constitutes a public health problem in Equatorial Guinea, positioning the country on the verge of achieving elimination. Additionally, the mapped prevalence of LF will facilitate the formulation of national strategies aimed at eradicating filariases countrywide.

**Graphical Abstract:**

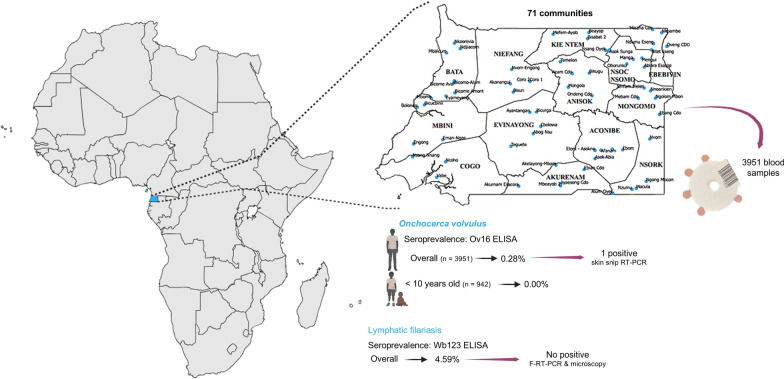

**Supplementary Information:**

The online version contains supplementary material available at 10.1186/s40249-024-01254-9.

## Background

Neglected tropical diseases (NTDs) are a group of diseases that place a constant and heavy burden primarily on the poorest, most marginalized, and isolated communities in the world. The African Region carries 39% of the global burden of NTDs – equating to over 580 million people [1]. Elimination of NTDs has recently emerged on the global health agenda and gained prominence with the release of the global plan to combat NTDs by the World Health Organization (WHO). In this sense, onchocerciasis and lymphatic filariasis (LF) specific targets are elimination (interruption of transmission) for the first and elimination as a public health problem for the later [2].

The success of vector control strategies alongside widespread mass drug administration (MDA) of ivermectin has significantly reduced the prevalence of onchocerciasis, making the transition from disease control to elimination achievable. Ivermectin distribution has been limited to areas with high onchocerciasis endemicity and loiasis free areas, due to the risk of severe adverse reactions in individuals with high-intensity *Loa loa* infections when treated with ivermectin [3]. Transitioning the focus of onchocerciasis programs from control to elimination necessitates a deeper insight into the infection’s geographic spread. Areas once excluded by control initiatives, deemed hypo-endemic or non-endemic, now require reevaluation to ascertain if onchocerciasis persists at levels sustaining transmission [4]. In this regard, serological assays and, to a certain degree, molecular diagnostics like PCR play a critical role, offering a more sensitive measure of infection or exposure.

Equatorial Guinea is endemic for onchocerciasis, LF, and loiasis, with notable regional disparities. Control efforts for onchocerciasis initially focused on Bioko Island, where loiasis was absent, starting with ivermectin mass treatments by the Onchocerciasis Control Program in West Africa (OCP) in 1990 [5]. This was followed by the African Program for Onchocerciasis Control (APOC) in 1995 implementing community-directed ivermectin treatments (CDTI) throughout the island by 2000 and large scale larvicide trials from 2001 to 2005 [6]. After 18 years of intervention, onchocerciasis has been largely eradicated from Bioko Island. However, the epidemiological situation remains unclear in mainland region. The latest APOC data from 2013 to 2015 show zero onchocerciasis prevalence based on skin snip biopsies. Previous nodule-based prevalence surveys conducted in 2008, categorised all communities as hypoendemic, except for 4 that were mesoendemic at the lower limit [7]. Besides, a systematic entomological survey conducted in 2019 did not found biting females of *S. damnosum* in the area (data from de Ministry of Health and Social Welfare of Equatorila Guinea, see supplementary file 1). Concerning LF transmission, a Rapid Assessment of the Geographical Distribution Bancroftian Filariasis (RAFIL) based mapping conducted in 2008 showed prevalence varies from 0 to 14% across the mainland region [8]. At the time this study was completed, no mass treatment against LT had been carried out in Equatorial Guinea. Meanwhile, loiasis affects 22–36% of the mainland population, as determined by the rapid assessment questionnaire (RAPLOA) [9].

This study aims to advance the mapping of onchocerciasis in mainland Equatorial Guinea as a critical step towards the goal of eliminating the disease at the national level. A secondary objective, in line with WHO’s integrative approach recommendations, is to evaluate the seroprevalence of LF within the same geographical area.

## Methods

### Study area

Mainland region of Equatorial Guinea comprises 4 provinces and 13 districts in a total area of 26,017 km², and is bordered on the north by Cameroon, on the east and south by Gabon, and on the west by the Atlantic Ocean. It has a population of 882,747 inhabitants according to the 2015 census, 72.2% of the whole country’s population (information from the National Onchocerciasis and Other Filariasis Control Programme). All the mainland districts are ivermectin-naïve and defined as *O. volvulu*s and LF co-endemic [5, 8, 10].

### Sample size and sampling strategy

From September to December 2019, a cross-sectional study was carried out to estimate the seroprevalence of onchocerciasis and LF in the continental of Equatorial Guinea.

The districts constituted the evaluation unit of the study. A minimum sample of 300 individuals per district was calculated based on WHO protocols [11]. The sampling frame was all communities within each district. The study purposefully sampled 3–5 first line communities in each district. All age groups were targeted. Within the selected districts, the location of the original first-line villages in relation to known breeding sites and distances between other first-line villages were verified. For the selection of communities to survey, we prioritized sites based on the following criteria:

- Communities identified with the presence of the aquatic stages of *S. damnosum* vector during the entomological survey conducted in February–March 2019 (supplementary file 1).
- To meet the quota of communities per district (*n* = 5), we included sentinel communities where a prevalence of nodules/skin snips greater than 10% was recorded in rapid mapping activities by APOC in 2008, 2013, and 2015 (personal communication with the Expanded Special Project for Elimination of Neglected Tropical Diseases, ESPEN).
- Should additional communities be needed, we first considered those with a prevalence below 10%, by rapid epidemiological mapping of onchocerciasis (REMO) or skin snip, selecting them in descending order of prevalence. Following this, communities identified with other *Simulium* species during the 2019 entomological study were selected.
- If more communities were still needed, we selected those located within 5 km of a risk zone, defined as areas adjacent to both sides of a river.

Within each community, a randomization system was established to cover approximately 60 members per community based on the community lists provided by the local authorities. Details on strategy sampling and community selection are provided in the Standard Operational Procedure SOP_01 (see supplementary file 2).

### Data and sample collection and management

Prior to starting the study, a comprehensive field training program was provided along with training on the proper use of sampling (see additional files 3, 4 and 5). The participation questionnaire was pre-tested one week before the beginning of the project.

All consenting individuals were asked for basic information about gender, age, the number of years lived in this village, travel history, and exposure to ivermectin and/or albendazole. Each participant received a unique, anonymized code assigned to a thick blood smear (TBS), filter paper disk for collecting dried blood spots (DBS) and to their questionnaires, ensuring confidentiality before sample collection.

Data and sample collections were carried out in a health post or a traditional house known as “house of the word” (see supplementary file 6). To this end, the members of the selected households were notified in advance, ensuring that everyone meeting the inclusion criteria was present on the day of the visit. The village or community chief took on the responsibility of summoning the participants.

The survey team also recorded basic information on each selected village including the associated district, village population, village global positioning system (GPS) coordinates, and identification of black flies by community leaders. Data were collected on smartphones and uploaded regularly to the electronic platform tailored by ESPEN Collect using a secure data kit software (SDK) [12]. Data were stored on a secure server. Field staff was previously trained in using these tools for data collection (see supplementary files 7 and 8). All data from questionnaires and samples were documented in notebooks and then input into a secure database by specialized data entry personnel. After the study concluded, DBS were dissociated from personal identifiers to ensure confidentiality.

For logistical reasons, all samples were taken during the day. Blood sampling was performed by fingerstick method collected onto Tropbio Filter Paper Disk and onto microscope slides. Following collection, filter paper disks were stored and transported to Madrid, Spain, for processing. Further details on sampling, sample collection, and transport are provided in the supplementary files 9, 10 and 11.

A follow-up visit was scheduled to collect samples from individuals who tested positive or had undetermined results for onchocerciasis. However, due to the COVID-19 pandemic, this visit was postponed to July 2021. During this visit, skin snip biopsies (see supplementary file 12) were taken from each iliac crest for PCR analysis, and peripheral blood samples were collected in EDTA tubes for serological retesting. Unfortunately, for lymphatic filariasis (LF), it was not possible to obtain a second nocturnal blood sample from individuals with positive or undetermined initial results.

### Microscopy laboratory testing

TBS were stained with 3% Giemsa solution for 30 min. TBS were used to calculate the *W. bancrofti* microfilaremia (microfilariae per milliliter of blood) considering that each TBS contained approximately 20 µl. Morphological identification was performed by two different expert microscopists. Every field on a slide was thoroughly examined before a negative result was determined.

### Serological laboratory testing

The blood samples collected on Tropbio Filter Paper Disk underwent analysis using two ELISA tests to detect specific IgG4 antibodies against the recombinant antigens Ov16 and Wb123 from *O. volvulus* and *W. bancrofti* respectively (Ov16-ELISA & Wb123-ELISA). Both recombinant antigens were obtained as previously described in Hernández-González et al. [13] and Herrador et al. [14].

The procedures for ELISA tests, including the controls used (including humanized monoclonal IgG4 against Ov16 antigen) [15], the calculation of the serological index (SI) and the interpretation of results, are detailed in the supplementary file 13.

#### Molecular laboratory testing

Skin snip biopsies and DBS were incubated at 56 °C overnight in ATL buffer previous to DNA extraction following the QIAamp^®^ DNA Mini Kit (QIAGEN GMBH, Germany) instructions. The presence of *O. volvulus* in the DNA from skin snip biopsies and *W. bancrofti* in the DNA isolated from blood samples were assayed using the F-RT-PCR described by Formenti et al. [16].

### Statistical analysis

Individual data and laboratory results were analyzed to obtain the frequencies of each variable. The prevalence of onchocerciasis and LF by district, along with 95% confidence intervals (*CI*) was determined using the method recommended by Brown et al. for interval estimation of a binomial proportion [17]. The software GeoDa was used for map representation.

## Results

In each of the 13 districts, over 300 participants were enrolled as part of the study population, totaling 3951 individuals examined across 71 communities, encompassing all districts (as detailed in the supplementary file 14). The median age of participants was 37 years, with an interquartile range (IQR) of 11–59 years. Females made up 55% of the study cohort. The breakdown of participants by sex, age group, and district is detailed in Table 1. The predefined sample size was successfully met in all districts. A local population’s lack of awareness and non-complaints regarding biting insects of *Simuliunm* spp. was registered during the survey.

**Table 1:** Characteristics of selected population, mainland Equatorial Guinea, 2019

Characteristics	Values	*n*	%
Sex	Female	2172	54.0
	Male	1779	45.0
Age group*, years	< 5	168	4.2
	5–9	767	19.4
	10–14	364	9.2
	15–29	415	10.5
	30–49	739	18.7
	50–69	1017	25.7
	> 70	480	12.1
District	Acurenam	303	7.7
	Akonibe	304	7.7
	Anisok	300	7.6
	Bata	303	7.7
	Ebebiyin	301	7.6
	Evinayong	302	7.6
	Kogo	303	7.7
	Mbini	319	8.1
	Micomiseng	301	7.6
	Mongomo	303	7.6
	Niefang	308	7.8
	Nsoknsomo	300	7.6
	Nsork	304	7.7

*Information on age was missing in one participant; *n*: number of participants; %: percentage.

### Onchocerciasis seroprevalence

**Fig. 1:**
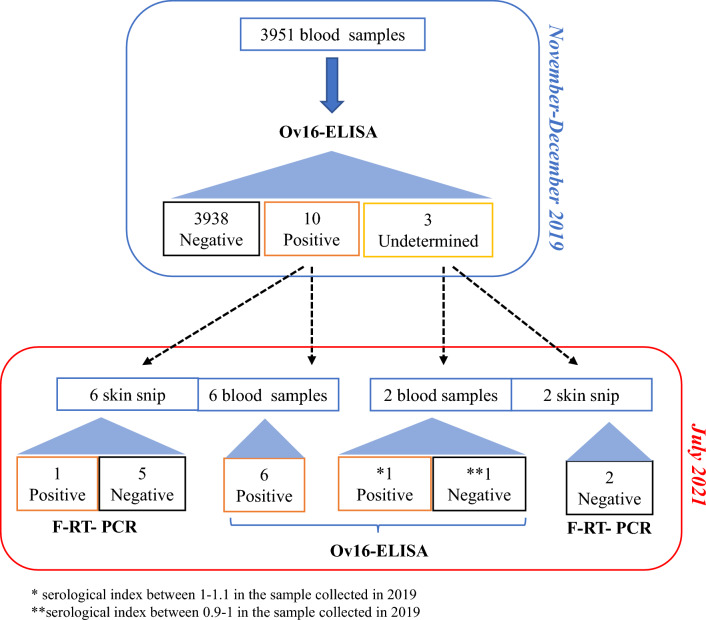
Flowchart of work and obtained results for onchocerciasis, continental Equatorial Guinea, 2019–2021

Out of 3951 participants, only ten tested positive for exposure to onchocerciasis using the Ov16-ELISA test, with an additional three samples classified as undetermined. Two years following the initial assessment, a second visit enabled the collection of blood samples and skin snip biopsies from eight of the previously identified positive and undetermined cases, due to their availability. The follow-up tests confirmed the serological positivity in all six initially positive individuals and in one case that was previously undetermined, as depicted in Fig. 1.

Microscopy examination of skin snip biopsies from all participants was negative for onchocerciasis, but DNA of *O. volvulus* was detected in one case, as confirmed by sequencing (Table 2). This individual was a 76-year-old male with recent travel history to Cameroon and no prior ivermectin treatment. Following the second sample collection, all seven serologically positive cases were over 50 years old (Table 2). Among those with positive or undetermined serology for onchocerciasis, nine (69%) reported past travel to Bioko Island, with all but one visit occurring over five years ago. Of the individuals who had never visited Bioko Island, three mentioned traveling to Cameroon (*n* = 2) or Gabon (*n* = 1). Regarding ivermectin intake, two seropositive cases and two with undetermined results reported using the medication. Co-infection with *L. loa* was observed in five individuals tested seropositive, as identified through TBS microscopy. Indeed, a 17% overall prevalence of *L. loa* was determined by TBS microscopy.

**Table 2:** Positive and undetermined cases in ELISA for onchocerciasis by district, mainland Equatorial Guinea, 2019–2021

Province / District	Gender	Age	Ov16 ELISA 1st visit	Reached 2nd visit	Ov16 ELISA 2nd visit	PCR Skin snip
Centro Sur /						
Evinayong	Female	81	Positive	Yes	Positive	Negative
Wele-Nzas/						
Mongomo	Male	51	Positive	No	··	··
Mongomo	Male	37	Positive	No	··	··
KieNtem						
Añisok	Female	53	Positive	Yes	Positive	Negative
	Female	21	UnD	No	··	··
Ebebiyín	Male	58	Positive	No	··	··
	Male	76	Positive	Yes	Positive	Positive
	Male	68	Positive	Yes	Positive	Negative
Micomeseng	Male	75	UnD	Yes	Positive	Negative
		90	Positive	Yes	Positive	Negative
Nsok-Nsomo	Male	50	Positive	No	··	··
	Male	54	Positive	Yes	Positive	Negative
	Female	31	UnD	Yes	Negative	Negative

*UnD* Undetermined

**Fig. 2:**
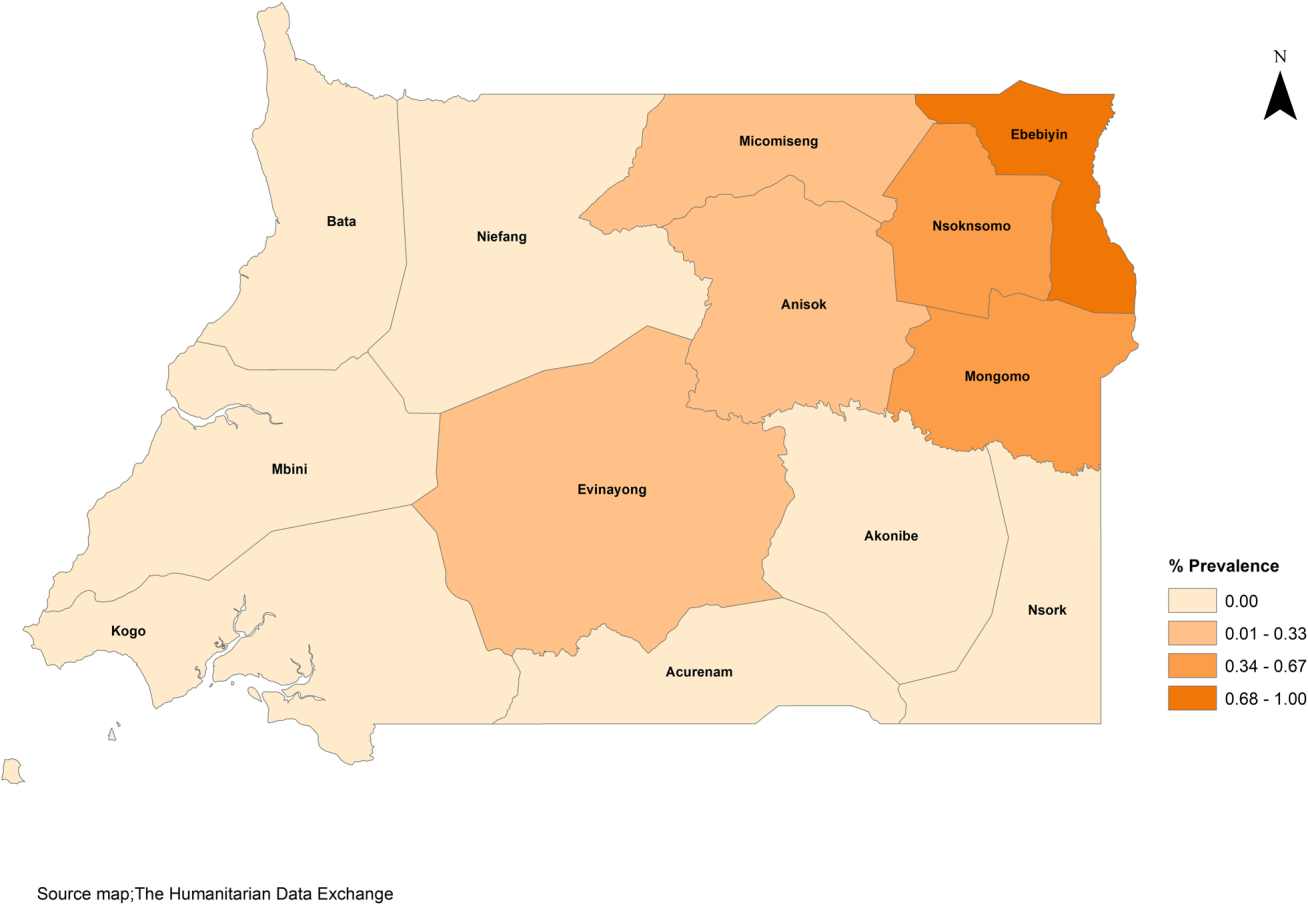
Seroprevalence of onchocerciasis (adult and infant population) by district in mainland Equatorial Guinea.

The majority of cases (9 out of 11) were identified in the northeast region of the continental area, specifically in the Kie-Ntem and Wele-Nzas provinces, with many residing in the same village. The districts with the highest seroprevalences were Ebebiyin, with a prevalence of 1.0% (95% *CI*: 0.2 to 2.9), followed by Nsok-Nsomo and Mongomo, each showing a prevalence of 0.7% (95% *CI*: 0.1 to 2.4). The overall seroprevalence calculated for the study was 0.3% (95% *CI*: 0.1 to 0.5), based on 11 seropositive ELISA results out of 3951 samples tested, as illustrated in Fig. 2 and detailed in additional file 15.

During the second visit, microscopy revealed no *O. volvulus* microfilariae in any of the eight skin samples collected. However, DNA was successfully amplified from one skin snip, and subsequent sequencing confirmed the presence of *O. volvulus*.

### Lymphatic filariasis seroprevalence

**Fig. 3:**
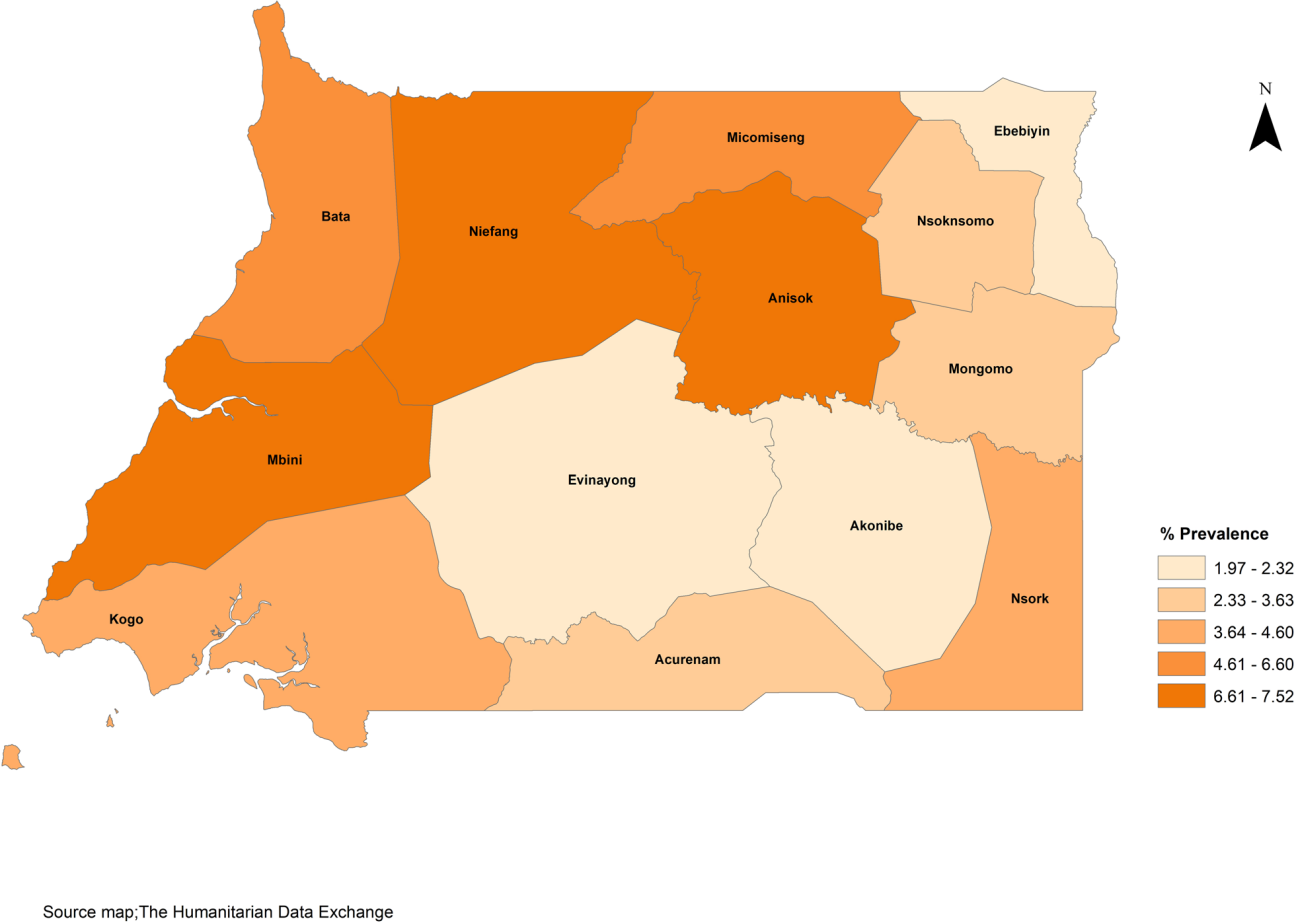
Seroprevalence of lymphatic filariasis (adult and infant population) by district in mainland Equatorial Guinea.

Overall, district seroprevalences ranged from 2.0% (95% *CI*: 0.8 to 4.0) to 7.52% (95% *CI*: 5.0 to 10.8), with a mean prevalence of 4.6% (95% *CI*: 4.0 to 5.3) (Fig. 3), details are displayed in Table 3. Out of 3951 samples, 182 tested positives for anti-Wb123 antibodies, suggesting exposure to *W. bancrofti*. Additionally, 84 cases were classified as undetermined, representing 2.4% of the sampled population.

**Table 3:** Overall and district seroprevalence of lymphatic filariasis

Province / District	Sample	Positive	UnD	Seroprevalence	95% *CI*
LITORAL					
Bata	303	20	6	6.6	4.2–9.9
Mbini	319	24	11	7.5	5.0–10.8
Kogo	303	12	8	4.0	2.2–6.6
CENTRO SUR					
Acurenam	303	11	7	3.6	1.9–6.2
Evinayong	302	7	5	2.3	1.0–4.5
Niefang	308	22	13	7.1	4.6–10.4
WELE-NZAS					
Akonibe	304	6	1	2.0	0.8–4.1
Mongomo	303	11	3	3.6	1.9–6.2
Nsork	304	14	9	4.6	2.6–7.4
KIE NTEM					
Anisok	300	21	10	7.0	4.5–10.3
Ebebiyin	301	7	5	2.3	1.0–4.5
Micomiseng	301	18	5	6.0	3.7–9.1
Nsoknsomo	300	9	1	3.0	1.5–5.4
All	3951	182	84	**4.6**	4.0–5.3

*UnD* Undetermined

Anti-Wb123 antibodies were found in the 5–15 year age group across all districts, except for Evinayong and Nsork. (Table 4). The seroprevalence rates in individuals over 15 were nearly twice as high, with variations by region. Among children, the districts of Mongomo (in Wele-Nzas) and Kogo (in Litoral) exhibited the highest seroprevalence rates, 8.1 (95% *CI*: 4.2 to 14.0) and 5.7 (95% *CI*: 2.1 to 12.1), respectively whereas Bata (also in Litoral), Niefang (in Centro Sur), and Anisok (in Kie Ntem) had the highest rates among adults, 8.6 (95% *CI*: 5.3 to 13.0), 8.4 (95% *CI*: 5.1 to 12.8), and 8.0 (95% *CI*: 4.8 to 12.5) respectively.

**Table 4:** Lymphatic filariasis seroprevalence by district and age, mainland Equatorial Guinea

District/ Province	< 15 years old		≥ 15 years old	
	Sample	Prevalence (95% *CI*)	Sample	Prevalence (95% *CI*)
LITORAL				
Bata	94	1.1 (0.6–5.1)	209	8.6 (5.0–13.0)
Mbini	97	1.1 (0.4–5.0)	222	6.3 (3.6–10.1)
Kogo	88	5.7 (2.1–12.1)	215	5.1 (2.7–8.7)
CENTRO SUR				
Acurenam	95	2.1 (0.3–6.8)	208	4.8 (2.5–8.4)
Evinayong	131	3.1 (1.0–7.2)	171	4.1 (1.8–7.9)
Niefang	105	N.A.	203	8.4 (5.1–12.8)
WELE-NZAS				
Akonibe	106	1.9 (0.3–6.1)	198	2.5 (0.9–5.5)
Mongomo	123	8.1 (4.2–14.0)	180	3.9 (1.7–7.5)
Nsork	85	3.5 (0.9–9.3)	219	6.4 (3.7–10.2)
KIE NTEM				
Anisok	101	4.0 (1.3–9.3)	199	8.0 (4.8–12.5)
Ebebiyin	116	4.3 (1.6–9.3)	185	1.6 (0.4–4.3)
Micomiseng	98	1.0 (0.4–44)	203	7.4 (4.4–11.6)
Nsoknsomo	61	N.A.	239	3.4 (1.6–6.2)
Total	1300	**2.9 (2.1–3.9)**	2651	5.5 (4.6–6.4)

*UnD* undetermined; *CI* confidence interval.

No *W. bancrofti* microfilariae were detected in any thick blood films (TBF). Furthermore, analysis for *W. bancrofti* DNA using F-RT-PCR conducted on all 3951 samples yielded negative results.

## Discussion

### Transmission levels of onchocerciasis in mainland Equatorial Guinea may fall below the thresholds deemed to pose a public health concern

This observation, derived from our mapping, is consistent with findings from the latest entomological study conducted in early 2019 (supplementary file 1). This earlier study suggested the absence of biting females from the *S. damnosum* complex, an observation further supported by our current data, which includes the local population’s lack of awareness and complaints regarding these insects. Historically, Equatorial Guinea participated in the APOC, where it was categorized as hypo-endemic for onchocerciasis [7]. The mainland region is also recognized as a West African focus for high loiasis prevalence [18], which has hindered the implementation of widespread ivermectin treatment—a critical obstacle to achieving the global goal of onchocerciasis elimination in Africa by 2030.

### WHO-recommended Ov16 based diagnostic methods confirmed previous APOC mapping results and, in line with latest entomological data, suggest a minimal or interrupted transmission

To determine the prevalence of onchocerciasis, our study employed serological tests in the first phase of the study, and serological and molecular methods, in the second phase. During the APOC period, onchocerciasis mapping utilized the REMO protocol which relies on nodule palpation [19]. This approach was applied in the 1999, 2008, and 2013 mapping exercises. In the 2013 survey, diagnostic techniques were expanded to include parasitological assessment by skin snip biopsy, which was then chosen as the primary method for the 2015 mapping exercise [7]. As the focus shifts from control to elimination, it is essential to reevaluate hypo-endemic regions using diagnostic tests that are both more sensitive and specific [4]. For this reason, our study employed the Ov16 ELISA test, adhering to the latest WHO guidelines, as it enables the detection of specific antibodies, that indicate infection status and parasite transmission in low-endemicity areas [11, 20]. There is an epidemiological consistency between 2013 and 2015 and our survey results, with zero microfilaremia prevalence in 2013/2015 and zero antibody prevalence below the age of 37 years of age in our survey. The quality and consistency of the serosurveillance data was assured by the use of a recombinant human IgG4 antibody to Ov16 in each assay. This inclusion ensures the establishment of standardized cutoffs and enhances the reproducibility of the technique across different laboratories and users [15], a practice that has been successful in previous onchocerciasis mapping efforts [14, 21].

### No mass ivermectin treatment is needed in the continental area

Our findings reveal a seroprevalence of 0.28%, representing a total of 11 positive cases—all adults aged between 31 and 90 years—out of 3951 individuals evaluated. This updated seroprevalence significantly falls below the biological threshold of 2% antibody prevalence in adults, which is used to determine the need for mass ivermectin treatment [22]. This decline in onchocerciasis prevalence, noted even without the implementation of MDA, mirrors trends observed in 2013. Although MDA with ivermectin has never been implemented in mainland Equatorial Guinea and was stopped on Bioko Island in 2016, 10.5% of participants in the current survey reported having received ivermectin treatment at least once in the past five years. This may reflect treatment for prior infections or possibly recall bias during the survey. Besides, the APOC program on Bioko Island has made great strides in onchocerciasis control in [16], which might have impacted the presence of onchocerciasis cases in the mainland region, as traveling between both areas is a common practice in the country. Unfortunately, detailed individual and contextual data that could shed light on these aspects are absent in the official documentation.

### Cross-border measures and entomological surveillance in specific areas are key to achieving the elimination of transmission.

The observed seroprevalence for onchocerciasis in our study was notably low, with a geographical cline of increasing prevalence from zero in the southwest to 0.68–1% in the northeast. Highest levels were detected in the province of Kie-Ntem, particularly in the Ebebiyin district, which borders onchocerciasis-endemic areas of Gabon and Moloundou in Cameroon. According to 2020 data from the ESPEN [23], there were 27 implementation units (IUs) identified as endemic for onchocerciasis in Gabon, yet none had commenced MDA. In contrast, Cameroon had achieved a program coverage of 45%, which increased to 73% according to the latest data available [24]. The dynamics of onchocerciasis control programs in neighboring countries are crucial, as the migration of human populations and black flies across borders significantly affects the effort to eliminate onchocerciasis [25]. While our study identifies cases primarily in border districts, we cannot definitively attribute these to cross-border movement; however, it remains a plausible explanation.

### The lack of detection of anti-Onchocerca antibodies in the tested infant population is consistent with minimal or interrupted transmission.

The zero-antibody prevalence below the age of 21 years of age in the current study, including 942 children under 10 years old, in our study suggests that onchocerciasis transmission is either nonexistent or occurs at very low intensity in mainland Equatorial Guinea, As WHO guidelines establish a sample size of 2000 children to detect a prevalence of less than or equal to 0.1% (at the upper bound of the 95% confidence interval) [26], we recommend conducting a new study focused exclusively on infant population to conclusively confirm the interruption of onchocerciasis transmission in the continental area. This study should be complemented by an entomological survey in the border areas where adult positives were identified, to provide comprehensive evidence supporting the cessation of transmission.

### Despite the observed decrease in seroprevalence, the implementation of a mass-specific treatment for lymphatic filariasis is still necessary.

Additionally, we assessed the seroprevalence of LF as a secondary objective, in alignment with WHO recommendations, optimize resource utilization and coordinate treatment strategies [27]. Equatorial Guinea, alongside neighboring countries, are historically recognized as endemic for LF [28]. Our findings indicate an overall LF prevalence of 4.6%, with higher rates observed in individuals over 15 years old (5.5%). Despite this, all samples tested negative for *W. bancrofti* through microscopy and F-RT-PCR analysis, noting that sample collection occurred during daytime. This prevalence is lower than the 7.3% rate among adults reported in the 2008 APOC survey, for the same districts, using the RAGFIL method [8]. Wb123 based ELISA was used for LF diagnosis instead of Filariasis Test Strip (FTS), therefore reducing the risk for cross-reaction with high-density *L. loa* infections [29]. In the past two decades, momentum to eliminate LF in Africa has significantly improved as a result of the development of single-dose treatment strategies, point-of-care diagnostic tools, and drug donations from pharmaceutical companies [30]. In Equatorial Guinea, these interventions have been delayed due to different reasons. MDA with albendazole twice a year has been implemented progressively since 2020, subsequent to the initial phase of our survey. Impact assessments of LF control strategies should be closely coordinated with the additional epidemiological and entomological surveys planned for onchocerciasis, maximizing the synergy between interventions for these co-endemic diseases.

The potential for pre-analytical bias arising from complex field logistics is acknowledged. To mitigate these biases, comprehensive guidelines and Standard Operational Procedures (SOPs), detailed in the supplementary files, were developed and trialed prior to initiating fieldwork. Furthermore, these SOPs underwent internal validation both before and throughout the field activities to ensure reliability and accuracy in data collection and analysis. In addition, the potential for cross-border transmission of onchocerciasis abovementioned, given the proximity of study areas to endemic regions of neighboring countries, could not be definitively assessed. This uncertainty may bias the understanding of local transmission dynamics.

## Conclusions

Bioko Island has recently satisfied the WHO criteria for the cessation of MDA, and post-treatment surveillance has since been implemented. Considering the latest findings from the mainland, achieving the elimination of onchocerciasis throughout Equatorial Guinea emerges as a viable objective. The current survey indicates an absence of onchocerciasis transmission in the continental region, rendering preventive chemoprophylaxis with ivermectin unnecessary. We recommend conducting a targeted onchocerciasis survey among the child population in the continental area, utilizing a sample size sufficient to confirm onchocerciasis is not a public health problem in this region. Concurrently, a comprehensive entomological and serological survey should be undertaken in border areas. Enhanced surveillance and NTD integrated mapping are recommended. Furthermore, establishing a synchronized approach among the onchocerciasis elimination committees of Equatorial Guinea´s neighbouring countries is essential to enhance coordinated cross-border elimination initiatives.

## Supplementary Information

Additional file 1: Conclusions of the report about *Simulium damnosum* situation in Equatorial Guinea, 2019.

Additional file 2: SOP _01_Sampling_Strategy.

Additional file 3: SOP _02_Work Field Daily Planning.

Additional filel 4: SOP _03_ Survey_conduct.

Additional file 5: SOP_04_Sampling preparation.

Additional file 6: SOP_05_ Weighted Random Sampling by Household.

Additional file 7: SOP_06_ Coordinates registration

Additional file 8: SOP_7_ ESPEN Collect_data_register.

Additional file 9: SOP_08_ Taking _Samples_Whatman.

Additional file 10: SOP_09_Thick_blood_smear_sampling.

Additional file 11: SOP_10_ Storage and Shipping.

Additional file 12: SOP_11_Skin_Snip.

Additional file 13: SOP_12_ OV16_Wb123_ELISA tests.

Additional file 14: Supplement Table 1. Participants enrolled by community and date of visit.

Additional file 15: Supplement Table 2. Onchocerciasis seroprevalence by district, mainland Equatorial Guinea from both visits.

## Data Availability

Most data generated or analysed during this study are included in this published article and its supplementary information files. 15 additional files have been prepared including 12 SOPs. Any other data used during the current study are available from the corresponding author on reasonable request.

## Abbreviations

APOC: African Program for Onchocerciasis Control
CDTI: Community-directed ivermectin treatments
*CI*: Confidence interval
DBS: Dried blood spots
ELISA: Enzyme-linked immunosorbent assay
ESPEN: Expanded Special Project for Elimination of Neglected Tropical Diseases
F-RT-PCR: Filaria-real time-PCR
FTS: Filariasis Test Strip
GPS: Global Positioning System
ISCIII: Instituto de Salud Carlos III
IQ: Interquartile range
IUs: Implementation units
LF: Lymphatic filariasis
MDA: Mass drug administration
MINSABS: Ministry of Health and Social Welfare
N: Number of participants
NTDs: Neglected tropical diseases
OCP: Control Program in West Africa
Ov16-ELISA: ELISA to detect specific IgG4 antibodies against the recombinant antigen Ov16 from *O. volvulus*
PCR: Polymerase test reaction
RAFIL: Rapid Assessment of the Geographical Distribution Bancroftian Filariasis
REMO: Rapid epidemiological mapping of onchocerciasis
SDK: Data kit software
SI: Serological index
SOPs: Standard Operational Procedures
TBS: Thick blood smear
UnD: Undetermined
Wb123-ELISA: ELISA to detect specific IgG4 antibodies against the recombinant
WHO: World Health Organization
