# Supplementary material for: Approaching onchocerciasis elimination in Equatorial Guinea: Near zero transmission and public health implication
Source: Infect Dis Poverty. 2024 Nov 14;13:86. doi: 10.1186/s40249-024-01254-9 (PMC11562331; doi:10.1186/s40249-024-01254-9)
Supplement: Supplementary file 1 — Additional file 1: Conclusions of the report about Simulium damnosum situation in Equatorial Guinea, 2019. [file 40249_2024_1254_MOESM1_ESM.docx]

**Conclusions of the report: SITUATION OF SIMULIUM DAMNOSUM SL LARVAE IN VARIOUS RIVERS IN CONTINENTAL EQUATORIAL GUINEA IN FEBRUARY/MARCH 2019**

Soungalo T, Coulibaly D, Nguema R, Boneho J, Mba E.

NATIONAL PROGRAMME TO COMBAT AGAINST ONCHOCERCIASIS AND OTHER FILARIASIS

Republic of Equatorial Guinea Ministry of Health and Social Welfare DIRECTORATE GENERAL OF HEALTH, PUBLIC HEALTH AND PLANNING

A survey of *Simulium damnosum* s.l. breeding sites was conducted during the first peak rainy season of 2019 (February - June). The survey covered the 13 Health Districts (HDs) of the continental region. A total of 109 watercourses were visited and 107 potential pre -imaginal breeding sites were investigated. Pre-imaginal forms of *S. damnosum* s.l. were found. The cytotaxonomic identification of the captured *S. damnosum s.l*. larvae was carried out by the WHO/ESPEN DNA Laboratory in Ouagadougou (Burkina Faso). The conclusions of the report are transcribed:

CONCLUSIONS

*“Our survey is representative only of the rainy season, and more specifically of the first rainy peak of 2019. It is also incomplete because it does not take into account scientific research into the imaginal populations, primarily biting females. The sites surveyed only concern part of the river system and are relatively sparsely populated. Furthermore, if we consider that at other seasons the breeding sites could be significantly more numerous and more widespread, and therefore more productive in terms of imagos, the statements made by the villagers on the absence of any nuisance from Simulium stings, if confirmed scientifically by standardised captures, would lead us to conclude that local Simulium damnosum sl females are not anthropophilic to any great extent, if not completely.*

*In the continental region there are pre-imaginal deposits of S. damnosum s. l. and the identification by cytotaxonomy of the larvae collected reveals that they belong 100% to the species Simulium mengense.*

*Despite the inadequacy of the investigations at the time of the survey, the absence of biting females of the Simulium damnosum complex is probable and corroborated by the lack of knowledge of these insects and their nuisance by the local population. This absence is probably related to the low productivity of the breeding sites, but a non-anthropophilic behaviour of the females cannot be ruled out a priori. These results, compared with those of the partial entomological observations from the 2013 survey, lead to the conclusion that there is no seasonal transmission of onchocerciasis in the region surveyed. Given the current state of knowledge, this region is not eligible for mass treatment with ivermectin.”*
