# Supplementary material for: Approaching onchocerciasis elimination in Equatorial Guinea: Near zero transmission and public health implication
Source: Infect Dis Poverty. 2024 Nov 14;13:86. doi: 10.1186/s40249-024-01254-9 (PMC11562331; doi:10.1186/s40249-024-01254-9)
Supplement: Supplementary file 2 — Additional file 2: SOP _01_Sampling_Strategy. [file 40249_2024_1254_MOESM2_ESM.docx]

**SOP _01_SAMPLING_STRATEGY**

- **SOP code:** SOP _01_Sampling_Strategy _v03_EN
- **Area:** Equatorial Guinea Mainland
- **Version:** V03
- **Language:** English
- **Title:** Operational procedures on sampling calculation and sampling strategy in schools
- **Written by /date:** Zaida Herrador, 14/10/2019
- **Revised by / date:** Laura Reguero and Marta García, 15/10/2019; Zaida Herrador 04/11/2019
- **Approved by / date and signature:** Agustín Benito 19/11/2019
- **Original version:** Spanish

1. **SAMPLE CALCULATION AND SAMPLING STRATEGY**

The total sample size is 3900 participants, 300 participants per district (sampling unit, n=13). All age groups will be represented, preferably 50% male.

Following WHO recommendations, five sentinel communities have been selected in each of the 13 existing districts in mainland GE (total 65 communities). The location will be verified by recording the coordinates of the sentinel communities. Specifically, those communities have been selected:

- Communities identified with the presence of the aquatic stages of *Simulium damnosum* vector during the entomological survey conducted in February-March 2019.
- In addition, to complete the number of communities to be visited in each district (n=5), sentinel communities where a nodule/ skin snips prevalence above 10% was recorded during the rapid mapping activities conducted by APOC will be included.
- In case the number of communities is still insufficient, (a) communities where the prevalence by REMO/skin snips is less than 10% shall be included first (in decreasing order), and (b) communities where other *Simulium* species have been identified during the entomological visit.
- Within 5 km of an area at risk (on both sides of a river)

The theoretical selected communities are shown in Figure 1S. In addition, a list of **alternative communities** has been prepared following the same criteria (to be visited in case the established sample size is not reached from the communities selected as first line).

**Figure 1_ SOP_01.- Location of the selected communities**


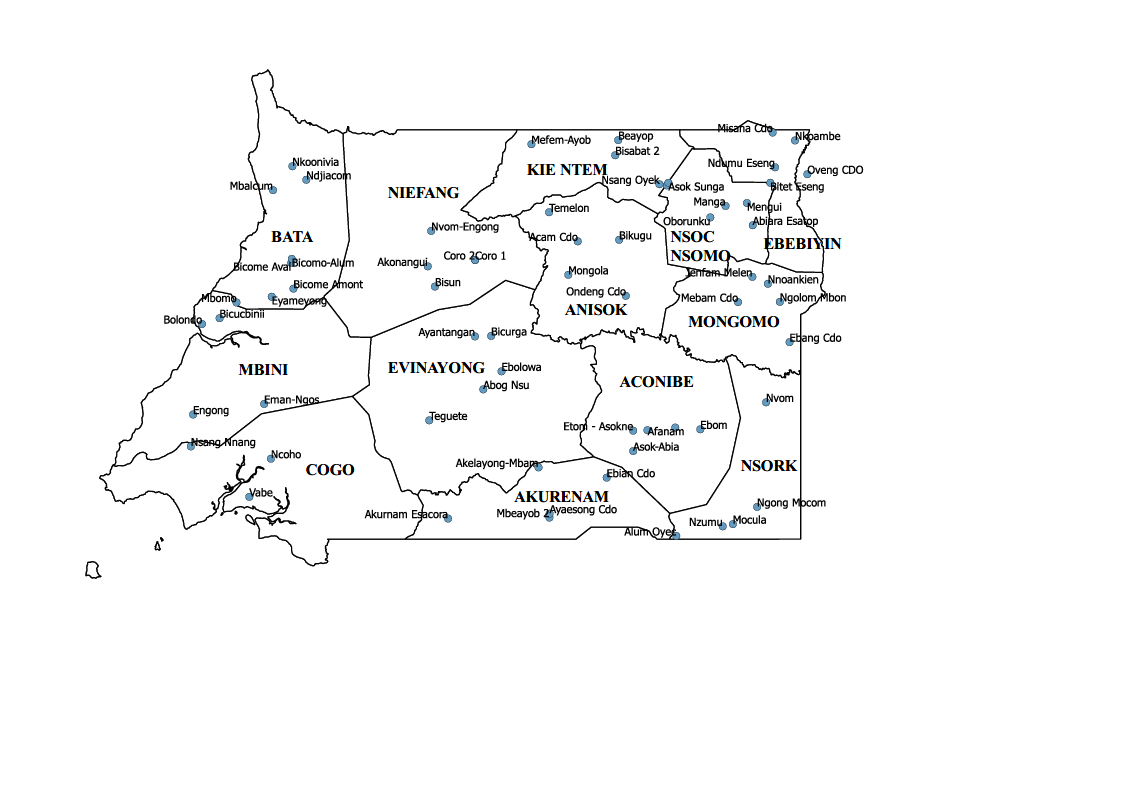


The visit will start in each district in those communities that have a smaller number of inhabitants > 5 years old (to estimate *a posteriori* in the larger communities how many more individuals would have to be included from that community to reach the sample unit size (n=300)).

- 1. **Sampling strategy**
- In each community, 13-15 households will be randomly selected (based on an expected number of inhabitants per household of 4-6).
- In addition, 5 alternative households will be selected, also randomly, to be used in case: a) they do not agree to participate; and/or b) the n=60 is not reached in that community.

All inhabitants of the randomly selected households (children and adults) will be offered to participate/include in the study.

In order to carry out this selection, the lists of households per community, which will have been collected during an exploratory visit in October, will be available in advance.

Sampling of the 60 participants in each community will take place at the health post or word house. For this purpose, the members of the selected households will be summoned in advance, so that on the day of the visit all those who meet the inclusion criteria will be present. The village or community head will be responsible for summoning participants (and alternative participants, in case of absence, not meeting the inclusion criteria, or not accepting participation in the survey).

The following table shows the ideal distribution of participants in a community (table 1_SOP_01)/district (table 2_SOP_01) by gender and age group:

**Table 1_SOP_01.** Age and sex distribution of participants from each of the communities (n=65)

| **Age groups** | **Male participants** | **Female participants** | **Total** |
| --- | --- | --- | --- |
| 5-9 years | 10 | 10 | 20 |
| 10-20 years | 10 | 10 | 20 |
| 20 years and older | 10 | 10 | 20 |
| **Total** | 30 | 30 | 20 |

**Table 2_SOP_01.** Age and sex distribution of participants in each of the districts (n=13)

| **Age groups** | **Male participants** | **Female participants** | **Total** |
| --- | --- | --- | --- |
| 5-9 years | 50 | 50 | 100 |
| 10-20 years | 50 | 50 | 100 |
| 20 years and older | 50 | 50 | 100 |
| **Total** | 150 | 150 | 300 |

While it is important to establish indicative quotas at the community level, the most important is to maintain representativeness at the district level.

1. **PROCEDURE FOR THE SELECTION OF HOUSEHOLDS**

**In those communities where there are less than 60 people meeting the inclusion criteria, no procedure will be necessary, as all of them will be included.**

In communities with more than 60, a random selection of households weighted by household size (population) will be made. Estimating an average of 4-6 participants per household, in total it will be necessary to select 10-15 households (select more in case of missing), although this number of clusters may vary depending on the total size needed (if there are communities with a small n, it will be necessary to complete the n from these larger communities).

- 1. **Random selection of households weighted by household size**

Procedure for household selection:

1. Construct an Excel with the names of households (heads of households) per community and the number of inhabitants (>5 years) per household.
2. In Excel, in a new column, we calculate the cumulative population density.
3. Then we calculate the sampling interval (total population/number of clusters (=households) that we have decided in advance we are going to select).
4. Then we calculate in Excel with the function random.between(x;y) a random number between 1 and the value of the sampling interval.
5. In the cumulative population column, we look for the interval in which our random number would fall.
6. We then add our sampling interval to this random number to obtain the second cluster. To this value we add the interval again to identify the next cluster, and so on.

This process will be done for each community before the visit, as soon as the lists are available. The president of the community will notify the households in advance so that on the day of the visit they can come to the health post/sword house for sampling.

- 1. **Criteria for inclusion in the study**
- Inclusion criteria: any resident of an eligible community within the UIs (district) who is 5 years of age or older.
- Exclusion criteria: persons with clinical or mental illnesses that are limiting to participation in the study or who cannot provide informed consent or obtain informed consent from a parent or guardian will be excluded (for boys and girls only).

1. **SUMMARY**

- Random selection of 3900 participants.
- Obtaining informed consents (in case of children under 15 years signed by parents/guardians).
- Piloting of the fieldwork (1 day) and revision of the SOPs/surveys according to the results of the piloting.
- Conducting the survey and taking samples.

1. **REFERENCES**

- WHO (2016). Guidelines for stopping mass drug administration and verifying elimination of human onchocerciasis: criteria and procedures. <http://apps.who.int/iris/bitstream/10665/204180/1/9789241510011_eng.pdf?ua=1>
