# Supplementary material for: Approaching onchocerciasis elimination in Equatorial Guinea: Near zero transmission and public health implication
Source: Infect Dis Poverty. 2024 Nov 14;13:86. doi: 10.1186/s40249-024-01254-9 (PMC11562331; doi:10.1186/s40249-024-01254-9)
Supplement: Supplementary file 3 — Additional file 3: SOP _02_Work Field Daily Planning. [file 40249_2024_1254_MOESM3_ESM.docx]

**SOP _02_** **WORK FIELD DAILY PLANNING**

- **SOP code:** SOP _02_Work Field Daily Planning_v02_EN
- **Area:** Equatorial Guinea Mainland
- **Version:** V02
- **Language:** English
- **Title:** Operational procedures on daily team preparation and daily team dynamics
- **Written by /date:** Zaida Herrador, 16/10/2019
- **Revised by / date:** Marta García and Laura Reguero 17/10/2019
- **Approved by / date and signature:** Agustín Benito 19/10/2019
- **Original version:** Spanish

# OBJECTIVE

To describe the daily work procedures to be carried out in the field, including the materials to be prepared/held each day by work team and the daily work to be performed.

# DEFINITIONS

**Work team:** Teams composed of 2 national programme technicians, 1 expatriate (coordinator or assistant) and a local supervisor. The technicians will also be trained to take biological samples (see corresponding SOP) and to fill in the different questionnaires.

There will be a total of 4 teams. The teams will travel in the same car in pairs. Prior to the start of the community visits, a training day and a piloting of the survey will be conducted with these teams to ensure that all teams use common concepts and procedures.

# EXECUTION DATE

### Drill: 24 and 25 October 2019

### Team training: 11 November 2019

### Survey piloting: 12 November 2019

### Fieldwork: 12 November-6 December 2019

# PROCEDURES

## Daily material to be kept by the teams

### Logistics

- Lunches during field visits.
- Petrol.

### Stationery

- Photocopies of surveys.
- Photocopies of informed consent forms.
- Lists of households, communities of neighbours and village councils with their chairpersons.
- Pens, markers, backpacks.
- Tags
- GPS/Mobile.

### Sampling materials

- Tablecloth;
- Lancets;
- Disinfecting alcohol;
- Cotton wool;
- Gloves;
- Tropbio filter paper disks
- Material for drying filter paper discs: 7 mm rods φ x 40-60 cm and Styrofoam or string and durable clips / clips (for drying filter paper discs)
- Portas (with matte band and without matte band);
- Case for holders;
- Lancet disposal container;
- Adhesive tape;
- Soakers;
- Preprinted barcode labels;
- 7x10 cm resealable plastic bags with silicagel;
- Large resealable plastic bags to hold all Tropbio discs from the same province (50-75 discs per bag);
- Permanent markers.

## Daily schedule

**07:40.Meet driver in Asonga (Bata)/Hotel.** First team meeting with supervisor, coordinator and expatriate staff.

- Check that all the materials are available.
- Check (if we are carrying) that the GPS batteries are charged and that the mobile phones are working and have batteries.

**08:00. Collect the rest of the equipment.**

**09:30*. Arrival at the community, presentation and preparation of the pick-up point (health post, word house, etc).**

**10:00-12:30. Start of the 1st part of the fieldwork.**

- Record the coordinates
- During this period, each team will take samples and survey 30 people (there will be a total of 2 teams in that community at the same time), so that by the end of this first visit, information will have been collected from all 60 participants in that community. While one technician is taking the samples, the other one is surveying.

**12:30-13:00. Second meeting with the supervisor and lunch break.**

- If necessary, discuss with the supervisor any doubts and logistical problems that may have arisen during the morning.

**13:00-13:30 Travel to the next community.**

**13:30-14:00***. **Arrival at the community, presentation, and preparation of the pick-up point (health post, house of the word, etc.).**

**14:00-16:30.** **Start of the 2nd part of the fieldwork.**

- Register the coordinates
- During this period, each team will take samples and survey 30 people (there will be a total of 2 teams in that community at the same time), so that by the end of this first visit, information will have been collected from all 60 participants in that community. While one technician is taking the samples, the other one is doing the survey.
- Place the samples in the correct storage location.
- When all the interviews are completed, and before handing the material to the supervisor, each team has to review the questionnaires to try to correct any mistakes that have been made.

**16:00-18:00 End of field work (depending on distances). The most important thing is to** get samples from 240 participants per day (60 per team).

**Once back in Bata/District Hotel**

- Check and save completed surveys and samples taken (whatman paper and bags).
- Review materials needed for the next day's work.
- Record data in ESPEN collect and/or database.
- If supervisors/coordinators consider it necessary, a meeting could be arranged to clarify some issues.

# RELATED DOCUMENTS:

- SOP_03_SURVEY CONDUCT.
- SOP_08_TAKING SAMPLES WHATMAN.
- SOP_09_TAKING SAMPLES THICK DROP.
- SOP_10_STORAGE AND SHIPPING SAMPLES.
