# Supplementary material for: Approaching onchocerciasis elimination in Equatorial Guinea: Near zero transmission and public health implication
Source: Infect Dis Poverty. 2024 Nov 14;13:86. doi: 10.1186/s40249-024-01254-9 (PMC11562331; doi:10.1186/s40249-024-01254-9)
Supplement: Supplementary file 4 — Additional filel 4: SOP _03_ Survey_conduct. [file 40249_2024_1254_MOESM4_ESM.docx]

**SOP _03_** **SURVEY_CONDUCT**

- **SOP code:** SOP _03_ Survey_conduct _v02_EN
- **Area:** Equatorial Guinea Mainland
- **Version:** V02
- **Language:** English
- **Title:** Operational procedures on the conduct of the survey
- **Written by /date:** Zaida Herrador, 16/10/2019
- **Revised by / date:** Marta García and Laura Reguero 17/10/2019
- **Approved by / date and signature:** Agustín Benito 19/10/2019
- **Original version:** Spanish

# OBJETIVES

To describe the procedures to be carried out during the survey.

# APLICABLE TO

Surveyors, data entry clerks, team supervisors, coordinators and assistant coordinators.

# DEFINITIONS

**Work team**: Teams composed of 2 national programme technicians, 1 expatriate (one expatriate coordinator and one assistant) and one local supervisor. The technicians will be trained to take biological samples (see corresponding SOP) and to fill in the different questionnaires.

There will be a total of 4 teams. In each team there will be one technician, in charge of collecting the blood samples, and another technician in charge of conducting the survey. Before starting the school visits, a training day and a piloting (one day) of the survey will be conducted with these teams to ensure that all teams use common concepts and procedures.

# SURVEY

The survey collects:

- Basic socio-demographic information.
- Travel history related to the disease.
- A couple of images are included to identify if a type of medication has been taken previously.
- The RAPLOA questionnaire, which consists of three questions. To answer the second question, the photo must be shown (each team will have one photo).
- A final section of observations where additional personal information considered to be of interest for the study will be collected.

# IMPLEMENTATION DATE

- Training of the teams: 11^th^ November 2019.
- Field work: 11^th^ November – 6^th^ December 2019.

# RESPONSIBILITIES

## Who will conduct the survey: One of the two technicians from each team?.

## Who will supervise the survey? The local supervisor and the expatriate coordinator.

## The local supervisor: will check that the survey has been carried out correctly.

## The expatriate coordinator: will be responsible for checking that the established quotas (minimum of 60 participants per community), by sex and age, are met.

## Both:

## They will check that the material is correct and available.

## They will check that the surveys are properly labelled (with the same labels as on the samples).

## They will be in charge of the daily collection and custody of the surveys.

# PROCEDURES

## Steps to be followed in conducting the survey.

## Check that the participant meets the inclusion criteria.

## Obtain the signature of the informed consent form. In the case of minors under 15 years of age, the father, mother or legal guardian will sign.

## Place the barcode sticker in the space provided for this purpose on the survey. CHECK THAT THE SAME NUMBER IS ASSIGNED ON THE SAMPLES (WHATMAN PAPER AND HOLDER) AND ON THE SURVEY TO THAT PARTICIPANT.

## Complete the survey in clear handwriting.

## Check that no questions are left unanswered.

## Inform the participant (or his/her relatives) that in case he/she is positive for any of the diseases studied, we will have to contact him/her again to treat him/her and to obtain other samples for confirmation.

## Thank him/her for his/her participation.

# MATERIAL REQUIRED FOR THE SURVEY

## MATERIAL RESOURCES

## Logistics

- Lunches during field visits.
- Petrol.

## Stationery

- Photocopies of surveys.
- Photocopies of informed consent forms.
- Lists of households, communities of neighbours and village councils with their chairpersons.
- Pens, markers, back packs.
- Labels.
- GPS/Mobile.

# CONTINGENCIES AND CONFLICTS OF INTEREST

In case of illness or absence of one of the enumerators or field technicians, the supervisor (local or expatriate) of the team concerned will be responsible for replacing this person as soon as possible or for modifying the timetable as long as the project deadlines are respected.

# RELATED DOCUMENTS

- SOP_01_SAMPLING STRATEGY.
- SOP_06_COORDINATES REGISTRATION.
