# Supplementary material for: Approaching onchocerciasis elimination in Equatorial Guinea: Near zero transmission and public health implication
Source: Infect Dis Poverty. 2024 Nov 14;13:86. doi: 10.1186/s40249-024-01254-9 (PMC11562331; doi:10.1186/s40249-024-01254-9)
Supplement: Supplementary file 5 — Additional file 5: SOP_04_Sampling preparation. [file 40249_2024_1254_MOESM5_ESM.docx]

**SOP _04_SAMPLING PREPARATION**

- **SOP code:** SOP_04_Sampling preparation_v02_EN
- **Area:** Equatorial Guinea Mainland
- **Version:** V02
- **Language:** English
- **Title:** Operational procedures regarding preparation of equipment for sample collection
- **Written by /date:** Zaida Herrador, 15/10/2019
- **Revised by / date:** Thuy-Huong Ta Tang, Laura Reguero and Marta García 16/10/2019
- **Approved by / date and signature:** Agustín Benito 19/10/2019
- **Original version:** Spanish

# OBJECTIVE

To describe the procedures for the work to be carried out in the field by the teams to preparare sampling.

# DEFINITIONS

**Work team**: Teams composed of 2 national programme technicians, 1 expatriate (coordinator or assistant) and a local supervisor. The technicians will also be trained to take biological samples (see corresponding SOP) and to fill in the different questionnaires.

There will be a total of 4 teams. Before starting the visits to the communities, a training day and a piloting of the survey will be carried out with these teams to ensure that all teams use common concepts and procedures.

# IMPLEMENTATION DATE

## Drill: 24^th^ and 25^th^ October 2019.

## Training of teams: 11^th^ November 2019.

## Survey piloting: 12^th^ November 2019.

## Fieldwork: 12^th^ November-6^th^ December 2019.

# PROCEDURES

## Composition of the work teams

In total, it will be necessary to carry out the study:

- 2 field technicians.
- 1 supervisor.
- 1 coordinator.
- 1 driver.

## Visits to the communities

- Arrival to the community.
- Meeting with the community leader.
- Identify and inspect the place where the work will be carried out (preferably health post/house of the word). Check that the place has:
  - Roof (in case of rain).
  - Good ventilation.
  - Sufficient privacy for participants and technicians.
  - Two tables (large enough to set up our workspace on).
  - Chairs.
- Prepare the tables to distribute the material.
- Set up and check that all the necessary material is present:
  - Tablecloth.
  - Lancets.
  - Alcohol for disinfection.
  - Cotton wool.
  - Gloves.
  - Tropbio® filter paper discs.
  - Material for drying filter paper discs: 7 mm rods φ x 40-60 cm and Styrofoam or string and durable clips / clamps (for drying filter paper discs).
  - Holders (with matte band and without matte band).
  - Case for holders.
  - Container for disposal of lancets.
  - Sponge.
  - Soakers.
  - Pre-printed bar-coded labels.
  - 7x10 cm self-seal plastic bags with silicagel.
  - Large resealable plastic bags to hold all Tropbio discs from the same province together (50-75 discs per bag).
  - Permanent markers.
- Estimated working time:
  - Each team will handle 30 participants from that community. The following procedures will be carried out:

1. Check that the participant meets the inclusion criteria.
2. Obtain informed consent.
3. While one technician obtains the samples, the other technician will conduct the survey.

- In a 9-hour day (7 hours of fieldwork + 2 hours of rest/travel) each team will survey and obtain samples from 60 participants (1 community). In total, 240 participants per day.

# RELATED DOCUMENTS

- SOP_01_DAILY FIELD WORK_PLAN.
- SOP_02_WORK FIELD DAILY PLANNING.
- SOP_03_SURVEY CONDUCT.
- SOP_08_TAKING SAMPLES WHATMAN.
- SOP_09_TAKING SAMPLES THICK DROP.
- SOP_10_STORAGE AND SHIPPING SAMPLES.
