# Supplementary material for: Approaching onchocerciasis elimination in Equatorial Guinea: Near zero transmission and public health implication
Source: Infect Dis Poverty. 2024 Nov 14;13:86. doi: 10.1186/s40249-024-01254-9 (PMC11562331; doi:10.1186/s40249-024-01254-9)
Supplement: Supplementary file 6 — Additional file 6: SOP_05_ Weighted Random Sampling by Household. [file 40249_2024_1254_MOESM6_ESM.docx]

**SOP _05_** **WEIGHTED RANDOM SAMPLING BY HOUSEHOLD SIZE**

- **SOP code:** SOP_05_ Weighted Random Sampling by Household Size_v02_EN
- **Area:** Equatorial Guinea Mainland
- **Version:** V02
- **Language:** English
- **Title:** Operational procedures related to weighted random sampling by household size
- **Written by /date:** Zaida Herrador, 15/10/2019
- **Revised by / date:** Thuy-Huong Ta Tang, Laura Reguero and Marta García 16/10/2019
- **Approved by / date and signature:** Agustín Benito 19/10/2019
- **Original version:** Spanish

1. **OBJECTIVE**

To describe the procedure for the selection of households:

1. **PROCEDURE**
2. Build an Excel file with the names of the households (heads of household) per community and the number of inhabitants (>5 years) per household.
3. In Excel, in a new column, calculate the cumulative population density.
4. Then, calculate the sampling interval (total population/number of clusters (=households) that we have decided in advance we are going to select).
5. Then calculate in Excel with the function RANDBETWEEN(x;y) a random number between 1 and the value of the sampling interval.
6. In the column of the accumulated population, look for the interval in which our random number will be included.
7. Then add to this random number our sampling interval to obtain the second cluster. To this value we will add the interval again to identify the next cluster, and so on.
8. **EXAMPLE: WEIGHTED SAMPLING IN BAHIR DAR SCHOOLS**

Once we have estimated the sample sizes, we are going to identify the clusters. Our schools have very different number of students. For that reason, we would give more chance to large schools that perform an important volume of students to be included in the sample, as you can see below with more detail.

In resume, we will get an ordered systematic sample with cumulative inclusion probabilities according to the school proportional weight.

**1º** We calculate the cumulative number of students (see the fourth column in the table).

**2º** Then we divide the total number (N=24788) between the number of chosen clusters (seven clusters)*. In this case, we are going to estimate 7 clusters for urban schools and 5 clusters for rural schools. 24788/7≈3541

**3º** We estimate a random number between 1 and 3541 (to estimate this number we use the syntax **=RANDBETWEEN**(**bottom**,**top**) in Excel 2003. This function returns a random integer number between the numbers you specify (Bottom is the smallest integer and Top is the largest integer RANDBETWEEN will return)). A new random integer number is returned every time the worksheet is calculated. In this case, we got **275** as the random number between 1 and 3541.

**4º** Now, we select the school where the student number **275** is included (looking for it in the column: “cumulative number of students”). The first cluster will be in the first school of our list (the third one in the total list for urban and rural schools). The selected schools are underlined in red in our table.

**5º** To get the next cluster, we add 275 and 3541. The student number 3816 is in the second school (the fourth one in the total list for urban and rural schools). We repeat the process until we’ll got the 7 clusters.

1ºcluster: 275

2ºcluster: 275+3541=3816

3ºcluster: 3816+3541=7357

4ºcluster: 7357+3541= 10898

5ºcluster: 10898+3541= 14439

6ºcluster: 14439+3541= 17980

7ºcluster: 17980+3541= 21521

**6º** The number of students by cluster is: 435 (our sample size)/7(number of clusters) ≈ 62 (6 clusters of 62 students and 1 cluster of 63 students).

* The more clusters you could select to be visited in Bahir Dar, the more representative your sample will be. It will depend on your own resources (time, number of people in the team…).
