# Supplementary material for: Approaching onchocerciasis elimination in Equatorial Guinea: Near zero transmission and public health implication
Source: Infect Dis Poverty. 2024 Nov 14;13:86. doi: 10.1186/s40249-024-01254-9 (PMC11562331; doi:10.1186/s40249-024-01254-9)
Supplement: Supplementary file 7 — Additional file 7: SOP_06_ Coordinates registration [file 40249_2024_1254_MOESM7_ESM.docx]

**SOP _06_** **COORDINATES REGISTRATION**

- **SOP code:** SOP_06_ Coordinates registration_v02_EN
- **Area:** Equatorial Guinea Mainland
- **Version:** V02
- **Language:** English
- **Title:** Operational procedures on how to register coordinates
- **Written by /date:** Zaida Herrador, 15/10/2019
- **Revised by / date:** Thuy-Huong Ta Tang, Laura Reguero and Marta García 16/10/2019
- **Approved by / date and signature:** Agustín Benito 19/10/2019
- **Original version:** Spanish

# OBJECTIVE

To describe the procedures related to coordinate registration.

# DEFINITIONS

**Latitude:** Angular distance from a point on the surface of the Earth to the parallel of the equator.

**Longitude:** A definable metric concept for geometric entities over which a distance has been defined.

# APPLICABLE TO

Technicians, team supervis, coordinators and coordination assistants.

# IMPLEMENTATION DATE

## Training of the teams: 11^th^ November 2019.

## Fieldwork: 12^th^ November to 6^th^ December 2019.

# PROCEDURES (coordinates will be collected with only one of the following devices: GPS/ MapsMe/ESPEN Collect)

## 5.1 GPS

1. **The coordinator/coordination assistant will check the batteries each day before going to the field.**
2. The coordination assistant will record the coordinates after arriving in the community, once the work has started (in a break-time). To do this, he/she will follow the following steps.
   1. Switch on the GPS.
3. To acquire GPS signal, go outside in an open area until the bars indicate that you have a signal:

+

1. To get the coordinates, Mark **waypoint----🡪 Save and edit----🡪Change location+**

+

1. The coordinates are: latitude (listed first) and longitude (listed second), both measured in decimal degrees (e.g. Abiara Esatop: 1.898278, 11.20111). Write down all the numbers that appear (do not record either the letter or the zeros before the numbers).
2. If the coordinates are very different from those in the list of communities (Annex 1) or from each other, check in **Settings ----🡪Units** that the distance is measured according to the metric system.

## Maps.me mobile application

- Install the free application on your mobile phone.
- Download the package for Equatorial Guinea and Malabo (just search on the GE map and click on it).
- Press the compass/arrow button.
- Press my position for 3-4 seconds.
- Press marker.
- The coordinates will be displayed. Write them down on the paper.

## ESPEN Collect application

See SOP_07_ESPEN COLLECT DATA REGISTER
