# Supplementary material for: Approaching onchocerciasis elimination in Equatorial Guinea: Near zero transmission and public health implication
Source: Infect Dis Poverty. 2024 Nov 14;13:86. doi: 10.1186/s40249-024-01254-9 (PMC11562331; doi:10.1186/s40249-024-01254-9)
Supplement: Supplementary file 8 — Additional file 8: SOP_7_ ESPEN Collect_data_register. [file 40249_2024_1254_MOESM8_ESM.docx]

**SOP _07_** **ESPEN Collect_DATA_REGISTER**

- **SOP code:** SOP_7_ ESPEN Collect_data_register _v02_EN
- **Area:** Equatorial Guinea Mainland
- **Version:** V02
- **Language:** English
- **Title:** Operational procedures on data entry via the ESPEN Collect Tool
- **Written by /date:** Lidia Redondo Bravo, 17/10/2019
- **Revised by / date:** Laura Reguero, Marta García and Zaida Herrador 18/10/2019
- **Approved by / date and signature:** Agustín Benito 12/11/2019
- **Original version:** Spanish

# OBJECTIVES

Describe the procedure for entering the data obtained from the participant surveys using the ESPEN Collect tool.

# TARGET GROUP

- Supervisors, coordinators and expatriate staff.
- Data recording will be carried out by expatriate coordinators and assistants.

# PROCEDURES

## Downloading and setting up the application

## The ESPEN Collect application is only available for Android devices. To obtain this application, go to the "Play Store" application on your mobile device.


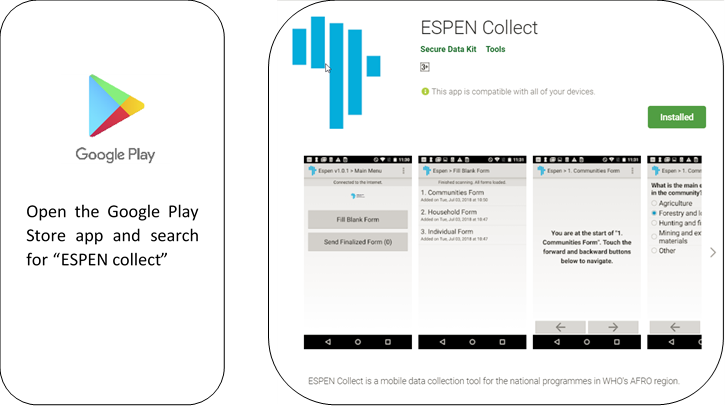


1. Once downloaded, the ESPEN Collect application will need to be configured for the DSA of a particular country. Each server URL, username and password will be unique for each country and DSA. To access the configuration, do the following:
   1. Open the ESPEN Mobile application by clicking on the ESPEN Mobile icon.
   2. Once the ESPEN Mobile application is open, you will see 3 dots stacked in the right corner, click on "Administrator Login".
   3. Enter the password and click OK.
   4. In the "Administration menu" click on the 3 dots stacked in the right corner again and click on "Change settings".
   5. You will need to enter the DSA settings for your country (this information will be provided to the administrators in each country). You will need to enter the following information:

- Server URL
- Username: collect
- Passsword:
  1. Once completed, click the "Back" button on your Android phone to return to the "Administration Menu"
  2. Once completed, click the "Back" button on your Android phone to return to the "Administration Menu"
  3. Click the "Get Blank Form" button and click "OK" in the Server Authentication box.
  4. Depending on the DSA, you will see a list of standardised surveys recommended for your DSA, click the checkbox next to each survey and click the "Get Selection" button at the bottom to download.
  5. Finally, click the "Back" button on your Android phone to return to the "Administration Menu". Click the "Back" button again to return to the main menu.

## Using the application for data entry

## Enter the ESPEN Collect application.

## You will then be presented with two options:

- 1. "Fill blank form" to start a new form.
  2. “Send finalised forms" to manually send those forms that have not been sent to the server automatically. When there are unsent forms, the number of unsent forms is shown in brackets (#).

## Clicking on "Fill in blank form" will open a screen showing all available forms.

## To start the new form, click on the name of the form. Each question must be answered before you can move on to the next one. No questions can be left blank. To move between questions, click on the arrows at the bottom of the question screen.

## Once the form is complete, a final page will appear showing all questions and answers. Check carefully that all answers have been answered correctly. If not, press the "Back" button and make the necessary corrections.

## Finally click on the "Save and submit form" button to exit.

The OEM project uses four survey files:

- Cluster: used to collect location data, such as GPS coordinates.
- Participant: used to collect demographic data of the individual.
- Rivers where breeding sites are located: This survey file should not be used during this part of the project.
- Community questions near breeding sites: this survey file should not be used during this part of the project.


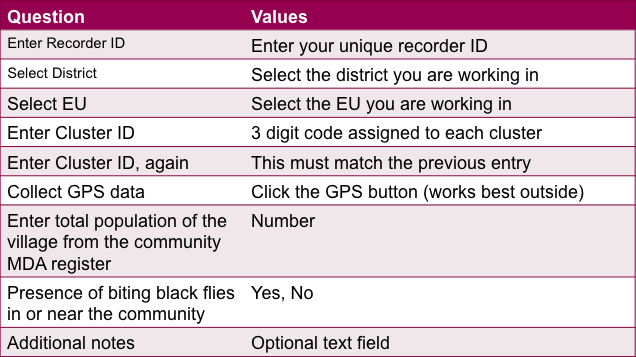


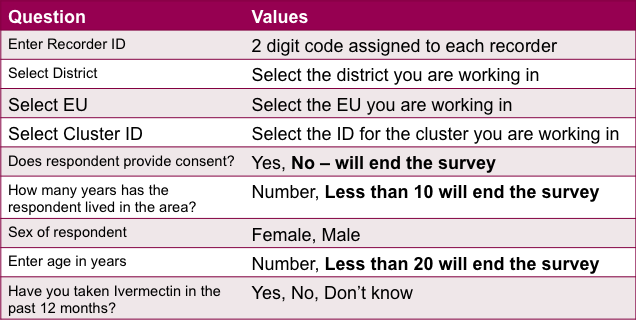


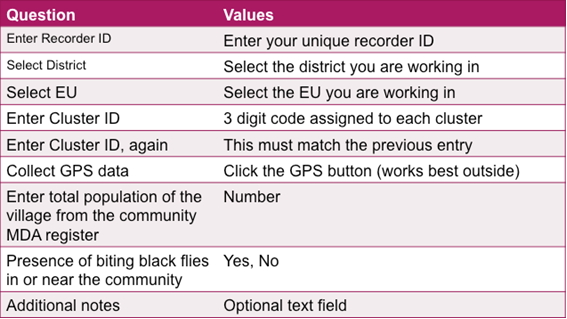


1. **Related documents**

- SOP_01_SAMPLING STRATEGY
- SOP_03_SURVEY CONDUCT
- SOP_08_TAKING SAMPLES WHATMAN
- SOP_09_TAKING SAMPLES THICK DROP
- SOP_10_STORAGE AND SHIPPING SAMPLE
- DSA Mobile Phone User Guide for Trainers (2019) Sightsavers.
- ESPEN Collect app Step-by-step guide <https://espen.afro.who.int/system/files/content/resources/ESPEN%20Collect%20app%20step%20by%20step%20guide_EN.pdf>

*Figures to prepare the SOP has been taken from the ESPEN Collect app Step-by-step guide.*
