# Supplementary material for: Approaching onchocerciasis elimination in Equatorial Guinea: Near zero transmission and public health implication
Source: Infect Dis Poverty. 2024 Nov 14;13:86. doi: 10.1186/s40249-024-01254-9 (PMC11562331; doi:10.1186/s40249-024-01254-9)
Supplement: Supplementary file 9 — Additional file 9: SOP_08_ Taking _Samples_Whatman. [file 40249_2024_1254_MOESM9_ESM.docx]

**SOP _08_** **TAKING _SAMPLES_WHATMAN**

- **SOP code:** SOP_08_ Taking _Samples_Whatman_V02
- **Area:** Equatorial Guinea Mainland
- **Version:** V02
- **Language:** English
- **Title:** Operational procedures on the collection and analysis of samples
- **Written by /date:** Thuy-Huong Ta Tang 15/10/2019
- **Revised by / date:** Zaida Herrador 16/10/2019; Lidia Redondo 16/10/2019; Laura Reguero 17/10/2019; Marta García 17/10/2019
- **Approved by / date and signature:** Agustín Benito 19/10/2019
- **Original version:** Spanish

# OBJECTIVES

To describe the procedure related to the collection of blood samples by capillary puncture on Tropbio filter paper discs (DBS: dried blood spot-dried blood spot), including drying and storage, and analysis of the samples.

# PRODUCT DESCRIPTION

**Tropbio Filter Paper disk**: The Tropbio filter paper disk (Tropbio Pty Ltd, QLD, Australia) for blood collection was originally designed as a WHO format for use with the Tropbio Og4C3 ELISA kit (*W. bancrofti*).

Blood collection in the field is made simple and convenient by taking blood by fingerstick on a filter paper disc. Once dried, samples can be easily transported to the laboratory for analysis or storage until analysis can be performed. This format is not only suitable for filarial work, but also for other parasites. The filter paper is packaged in 100 sheets x 4 discs/sheet, a total of 400 discs. Each disc has six circular protrusions (lobes or petals), and each lobe is designed to absorb approximately **10 μl** of blood. It measures 5 cm in diameter and the central circle is 7 mm φ.

# APPLICABLE TO

Technicians, team supervisors, coordinators and coordination assistants.

# IMPLEMENTATION DATE

## Team training: November 2019.

## Field work: November-December 2019.

# PROCEDURE

## Materials needed

• Lancets.

• Cotton wool and ethanol.

• Gloves.

• Sharps container.

• TropBio filter paper.

• Pre-printed barcode labels.

• 7 mm sticks φ x 40-60 cm and Styrofoam or string and durable clips/clips (for drying filter paper discs).

• 7x10 cm self-seal plastic bags.

• Large resealable plastic bags to put together all Tropbio discs from one province (50-75 discs per bag).

• Silica gel desiccant.

• Permanent markers.

**Important information:**

- Always wear gloves when handling filter paper and blood.

- One lancet per person.

- Two discs per individual (a total of **nine** blood lobes per individual).

## Preparation of the patient

The person taking the blood sample should wear clean, disposable gloves.

- Wipe the finger to be pricked with a cotton swab soaked in alcohol and allow it to dry. It is recommended to prick a finger on the non-dominant hand.
- Massage the finger a little. Prick the inner/medial side of the finger with a sterile lancet.

Dispose of the used lancet in a sharps container (use each lancet only once!)

## DBS Sampling and Storage

Each filter paper disc is labelled with the corresponding pre-printed barcode or marked with a permanent marker. Be careful not to cover the central circumference with the label - only one barcode number is used per person! Bring the six circular lobes close to the drop of blood on the finger. Make sure that each lobe is completely saturated with blood (no visible white spots on the lobe).


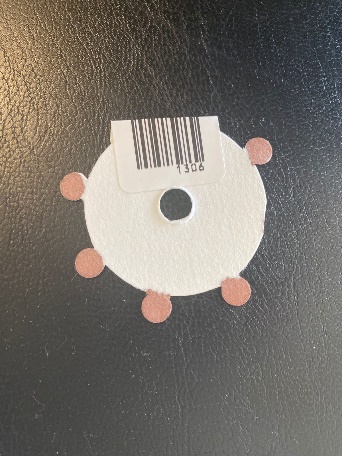


Source: self-created

Carefully place the filter paper disc on a 7 mm diameter rod inserted into a Styrofoam holder or secure the disc with a durable thread to dry. **Filters should dry at room temperature for at least 2 hours or overnight (overnight is best for complete drying.**


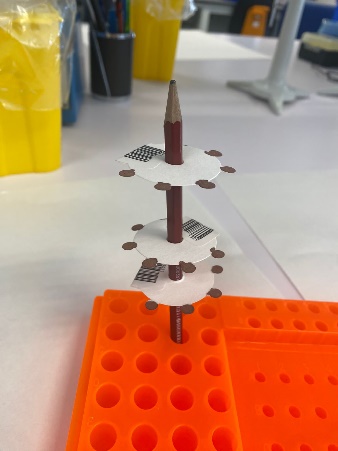


Source: self-created

Note: To avoid cross-contamination, make sure that the wet filter paper discs do not come into contact with each other. Do not place more than 3 wet filters on a single pencil. A small fan can be used to speed up the drying process.

After each filter disc is completely dry, make sure that each small sample bag is completely sealed so that the filters remain secure and insulated inside their individual bags with silica gel desiccant.


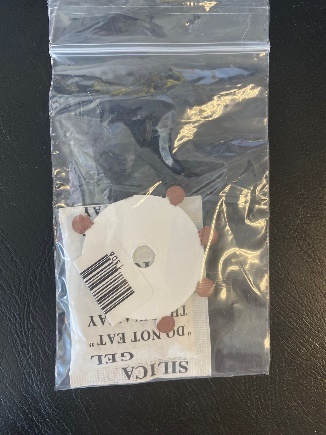


Source: self-created

1. **STORAGE OF FILTER PAPER DISCS**
   - For short-term storage (up to one week): store at 4°C in the plastic bags.
   - For long-term storage: store the bags at -20°C.
2. **RELATED DOCUMENTS**

- SOP_01_SAMPLING STRATEGY.
- SOP_03_SURVEY CONDUCT.
- SOP_10_STORAGE AND SHIPPING SAMPLE.
