# Supplementary material for: Approaching onchocerciasis elimination in Equatorial Guinea: Near zero transmission and public health implication
Source: Infect Dis Poverty. 2024 Nov 14;13:86. doi: 10.1186/s40249-024-01254-9 (PMC11562331; doi:10.1186/s40249-024-01254-9)
Supplement: Supplementary file 10 — Additional file 10: SOP_09_Thick_blood_smear_sampling. [file 40249_2024_1254_MOESM10_ESM.docx]

**SOP _09_THICK_BLOOD_SMEAR_SAMPLING**

- **SOP code:** SOP_09_Thick_blood_smear_sampling_V02_EN
- **Area:** Equatorial Guinea Mainland
- **Version:** V02
- **Language:** English
- **Title:** Operational procedures on the thick blood smear collection and analysis of samples
- **Written by /date:** Thuy-Huong Ta Tang 15/10/2019
- **Revised by / date:** Zaida Herrador 16/10/2019; Lidia Redondo 16/10/2019; Laura Reguero 17/10/2019; Marta García 17/10/2019
- **Approved by / date and signature:** Agustín Benito 19/10/2019
- **Original version:** Spanish

# OBJETIVES

To describe the procedure related to the collection of a sample to perform a thick blood smear for the parasitological diagnosis of microfilariae and their species identification.

# DEFINICIONES

**Thick blood smear (TBS):** thick layer of erythrocytes without haemoglobin (lysates). Blood elements and parasites, if present, are more concentrated than in an equivalent area of a thin smear. Thus, the thick smear is more efficient for the detection of parasites (increased sensitivity).

# APPLICABLE TO

Technicians, team supervisors, coordinators and coordination assistants.

1. **IMPLEMENTATION DATE**
   - Team building: November 2019
   - Field work: November-December 2019.
2. **PROCEDURES**
   1. **Sample collection: Thick blood smear (TBS)**

Identify the sample by sticking the corresponding barcode on the slide with matte edge.

The person taking the sample should always wear gloves.

- Hold the patient's hand palm upwards and wipe the third fingertip with a cotton swab soaked in 70% alcohol. In the case of a child, a heel swab can be used.
- Dry the finger with a clean, dry cotton swab while holding it vigorously. With a sterile lancet, quickly prick the fingertip laterally.
- Discard the sterile, single-use lancet in an appropriate container.
- Obtain a drop of blood by pressing lightly on the finger and deposit it on the slide with a clean, grease-free, matte edge and avoid touching the slide to the finger.
- Once the drop of blood has been deposited on the slide, using the corner of another slide (without a dull edge), spread the blood to a diameter of about 1 cm in a circular motion. The thickness should be such that it is possible to read through the preparation.
- The thick blood smear drop IS NOT FIXED with methanol or heat. Allow to AIR DRY protected from dust and insects, normally 30-60 minutes.
- Once dry, stored it in the slide holder.

1. **RELATED DOCUMENTS**

- SOP_01_SAMPLING STRATEGY
- SOP_03_SURVEY CONDUCT
- SOP_10_STORAGE AND SHIPPING SAMPLE
