# Supplementary material for: Approaching onchocerciasis elimination in Equatorial Guinea: Near zero transmission and public health implication
Source: Infect Dis Poverty. 2024 Nov 14;13:86. doi: 10.1186/s40249-024-01254-9 (PMC11562331; doi:10.1186/s40249-024-01254-9)
Supplement: Supplementary file 11 — Additional file 11: SOP_10_ Storage and Shipping. [file 40249_2024_1254_MOESM11_ESM.docx]

**SOP _10_ STORAGE AND SHIPPING**

- **SOP code:** SOP_10_ Storage and Shipping_v02
- **Area:** Equatorial Guinea Mainland
- **Version:** V02
- **Language:** English
- **Title:** Operational procedures on storage and shipment
- **Written by /date:** Zaida Herrador 21/10/2019
- **Revised by / date:** Belén García 23/10/2019, Thuy-Huong Ta 25/10/2019, Marta García 25/10/2019
- **Approved by / date and signature:** Agustín Benito 19/10/2019
- **Original version:** Spanish

# OBJECTIVES

To describe the procedures related to the storage and shipment of surveys and samples (DBS-dried blood spot and slides.

# DEFINITIONS

**Surveys:**

1. Individual.
2. Community.
3. Informed consent (IC).

**Samples:**

1. Samples collected on Whatman paper (DBS).
2. Thick blood smear in slides.

# APPLICABLE TO

Team supervisors, coordinators and assistant coordinators.

1. **IMPLEMENTATION DATE**

## Training of the teams: 11^th^ November 2019.

## Fieldwork: 12^th^ November to 6^th^ December 2019.

# PROCEDURES

## Surveys

1. All surveys from a community will be collected together and also together with the population survey of that community.
2. After the visit, and upon arrival at the hotel/Asonga, the coordinator and the expatriate team will sort and group the surveys by community, separating the surveys (along with the informed consents), into envelopes.
3. The envelope will be labelled with the name of the community, the range of numbers identifying the individuals in the community and the corresponding district.
4. Custody of the surveys + ICs is the responsibility of the expatriate coordinator.
5. The surveys will be transported to Spain in the suitcases of the coordinator + expatriate staff.

## Samples

### **Thick blood smear and slides**

1. Slides shall be stored, after checking they are dry, in the filing cabinet (see SOPs for sample handling).
2. This folder should be labelled and stored at the ISCIII office in Bata. After the study is completed, it should be sent to the National Centre for Tropical Medicine (CNMT).
3. The person in charge of the shipment in Guinea is the project coordinator with the help of the logistician and administrator.
4. Shipping address: Jose Miguel Rubio, Centro Nacional de Microbiología, Instituto de Salud Carlos III, Ctra. Majadahonda-Pozuelo km2, 28220 Majadahonda, Spain.
