# Supplementary material for: Approaching onchocerciasis elimination in Equatorial Guinea: Near zero transmission and public health implication
Source: Infect Dis Poverty. 2024 Nov 14;13:86. doi: 10.1186/s40249-024-01254-9 (PMC11562331; doi:10.1186/s40249-024-01254-9)
Supplement: Supplementary file 12 — Additional file 12: SOP_11_Skin_Snip. [file 40249_2024_1254_MOESM12_ESM.docx]

**SOP _11_ STORAGE AND SHIPPING**

- **SOP code:** SOP_11_Skin_Snip _v02_EN
- **Area:** Equatorial Guinea Mainland
- **Version:** V02
- **Language:** English
- **Title:** Operational procedures on skin_snip
- **Written by /date:** Laura Moya 03/10/2016 Adapted by Zaida Herrador 17/05/202
- **Revised by / date:** Thuy-Huong Ta Tang, José Miguel Rubio and Policarpo Ncogo Belén García 24/05/2021
- **Approved by / date and signature:** Agustín Benito 15/05/20
- **Original version:** Spanish

1. **OBJECTIVE**

To describe how to perform a skin snip.

1. **DEFINITION**

The skin snip consists of performing a biopsy of exsanguinated skin to detect microfilariae in the dermis.

1. **PROCEDURE**
   1. Put on gloves before starting the process (it is better to start with the oldest subjects.
   2. Use a cotton wool pad to apply alcohol to the area of skin to be pricked (iliac crest).
   3. The biopsy is performed using a sclero-corneal punch (Walser forceps) or by lifting a small cone of skin 3 mm in diameter with a needle and then shaving it with a scalpel.
   4. Two exsanguinated skin samples shall be taken, one from each iliac crest (one sample for microscopy and one for PCR).
   5. Each sample shall be placed in a PCR tube allocated for that patient, which shall contain 50-100 µl of saline.
   6. The corresponding part of these two samples shall be sealed with parafilm paper and the material corresponding to each patient and the plastic bag containing eachpatient's material shall be duly coded with a permanent marker pen. Use the ID of the first survey for coding.
   7. Disinfect the used biopsy material properly with the steriliser assigned for this purpose (in case Walser forceps have been used). If individual needles and scalpels are used, they are discarded, and new ones are used for another patient.
   8. Dispose of the waste in the corresponding plastic tank assigned for this purpose.
   9. The tissue corresponding to the iliac crest samples shall be incubated for 24 h at room temperature to allow the microfilariae to emerge from the dermis.
   10. After this time, the samples will be placed in a holder with a cover slip and the corresponding reading will be done with an optical microscope at the central laboratory in Malabo. During the field work, the samples shall be kept cold in a refrigerator (IMPORTANT: DO NOT FREEZE).
   11. After reading by microscopy, even if negative, put the skin pinch back in the eppendorf to send it to Madrid. Label the pinch that has been seen by microscopy to check for contamination by handling.
   12. Subsequently, they will be sent (the two skin pinches) to the National Microbiology Centre for DNA extraction and molecular analysis. Until shipment, samples shall preferably be kept in a refrigerator.


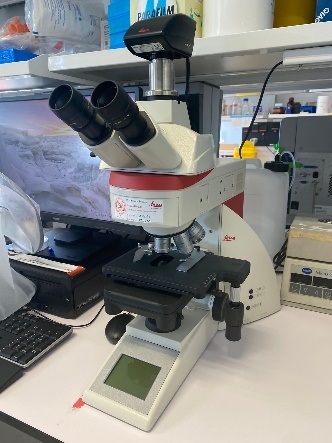

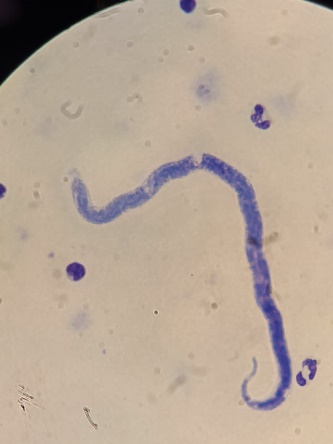


Source: self-created

# RELATED SOPS

- SOP_010_ Storage and Shipping_v02
