# Supplementary material for: Approaching onchocerciasis elimination in Equatorial Guinea: Near zero transmission and public health implication
Source: Infect Dis Poverty. 2024 Nov 14;13:86. doi: 10.1186/s40249-024-01254-9 (PMC11562331; doi:10.1186/s40249-024-01254-9)
Supplement: Supplementary file 13 — Additional file 13: SOP_12_ OV16_Wb123_ELISA tests. [file 40249_2024_1254_MOESM13_ESM.docx]

**SOP _12_ OV16_Wb123_ELISA tests**

- **SOP code:** SOP_12_ OV16_Wb123_ELISA tests_v02_EN
- **Area:** Equatorial Guinea Mainland
- **Version:** V02
- **Language:** English
- **Title:** Operational procedures to perform OV-16 and Wb123 ELISA tests
- **Written by /date:** Ana Hernández-González 18/09/2019
- **Revised by / date:** María Jesús Perteguer 19/09/2019
- **Approved by / date and signature:** Agustín Benito 23/09/2019
- **Original version:** Spanish

# OBJECTIVES

To describe the procedure of the ELISA tests with the recombinant antigens OV-16 and Wb123 for the detection of specific circulating antibodies in blood collected on Tropbio Filter Paper disks.

# PRODUCT DESCRIPTION

OV-16 in an ELISA format has been used to establish the status of infection and parasite transmission by detecting specific antibodies in areas of low endemicity. While Wb123-ELISA has been used in seroprevalence studies. Both are indirect ELISA tests, based on the detection of IgG4 antibodies in serum or blood against *Onchocerca volvulus* recombinant antigen OV16 and *Wuchereria bancrofti* recombinant antigen Wb123 as markers of exposure to onchocerciasis and lymphatic filariasis.

# APPLICABLE TO:

Technicians, team laboratories supervisors.

# IMPLEMENTATION DATE

- Training: September-November 2019.
- The procedures were carried out once the samples were received in the laboratory:

3953 samples corresponding to the first field work. Dates: July 2020-February 2021.

8 samples corresponding to the second field work. Dates: February 2022.

# PROCEDURE

## Materials needed

- Scissor and tweezers.
- Flat-bottom plates Nunc MediSorp® (Thermo Scientific, Denmark).
- 4ºC chamber.
- Incubator.
- Wilmut tubes for elution of blood.
- Wilmut tubes racks.
- Plate shaker.
- Automated ELISA analyzer (Dynex DSX System, 1DXC-1100).

## Reagents needed

- Recombinant antigens OV-16 and Wb123.
- PBS.
- Tween 20.
- Skimmed milk poder.
- Stabilcoat Immunoassay Stabilizer reagent (Sigma Aldrich, MO, USA).
- Positive and negative controls.
- Conjugated antibody: mouse anti-human IgG4 antibody labeled with peroxidase (SouthernBiotech, AL, USA).
- KPL Sureblue™ TMB Microwell Peroxidase Substrate.

## ELISA tests

*Coating*

- The purified recombinant antigens are placed on separate 96 wells flat-bottom plates Nunc MediSorp® (Thermo Scientific, Denmark) at 0.5 µg/ml and 5 µg/ml for the rOV16 and rWb123 respectively in carbonate buffer pH 9.6.
- The plates are covered and hold at 4°C overnight.
- The day after, the wells are washed with 300 µl of PBS-Tween 0.3% for three times.
- Plates are treated with Stabilcoat Immunoassay Stabilizer reagent (Sigma Aldrich, MO, USA) according to the manufacturer's recommendations, and store at 4°C in opaque airtight envelopes with desiccant until use.

*Samples and controls*

- **Samples**
- A single dried blood ear is excised from the Tropbio Filter Paper Disk using tweezers and a scissor.
- The blood is eluted in 500 µl of PBS-Tween 0.3% buffer with 5% skim milk overnight at 4°C on an orbital shaker.
- 100 µl of each eluted sample is placed in duplicate in the OV16 and Wb123 plates.
- **Controls**
- The OV16-ELISA includes a standard curve made from the dilution of a humanized monoclonal IgG4 antibody against OV16 antigen (AbD19432_h_IgG4_Pro Anti-recOV16-GST) (BioRad, Germany) as a positive control. The monoclonal is diluted from 200 ng/ml to 0.2 ng/ml in PBS-Tween 0.3% buffer with 5% skim milk to generate a standard curve used in all the plates.
- The Wb123-ELISA test includes a standard curve made from a *W. bancrofti* positive sera pool. The pool is diluted from 1/1600 to 1/7500 in PBS-Tween 0.3% buffer with 5% skim milk.

The samples and the standard curves are incubated in the corresponding plate for an hour at 37°C.

*Conjugated antibody*

The plates are placed in an automated ELISA analyzer (Dynex DSX System, 1DXC-1100) where the remaining steps are carried out. The running program includes washing steps and two more incubation steps as follows:

- The wells are washed with 300 µl of PBS-Tween 0.3% for five times.
- Next step is adding 100 µl/well of a mouse anti-human IgG4 antibody labeled with peroxidase (SouthernBiotech, AL, USA) at a 1/20000 dilution for the OV16-ELISA and 1/10000 for the Wb123-ELISA.
- The plates are incubated at 37°C for an hour.

*Developing and final read*

- After incubation, the wells are washed again with 300 µl of PBS-Tween 0.3% for five times.
- 100 µl/well of KPL Sureblue™ TMB Microwell Peroxidase Substrate (Seracare Life Sciences, MA, USA) are dispensed.
- The OV16-ELISA plates are kept for 40 minutes at room temperature in the dark and the Wb123-ELISA plates for 30 minutes.
- The reaction is stopped with 0.5 M sulphuric acid.
- Optical densities corresponding to each well are read at 450 nm.

*Interpretation of results*

OV16-ELISA test: the cut-off was already set at the dilution 2 ng/ml.

Wb123-ELISA test: the cut-off is the optical density OD corresponding to the dilution 1/5000 for our positive control.

Serological indexes (SI) are calculated as following: the mean OD obtained for each sample is divided by the mean OD value of the cut off point for each assay.

$$\frac{OD sample}{OD mAB 2 ng/ml}$$

$$\frac{OD sample}{OD pool dilution 1/5000}$$

Results above 1.1 were considered positive, values below 0.9 negative, and values between 0.9-1.1 undetermined.

**Important information:**

- Always wear gloves when handling filter paper and blood.

# RELATED DOCUMENT AND REFERENCES

- SOP_08_SAMPLE_TAKING_WHATMAN
- Golden A, Stevens EJ, Yokobe L, Faulx D, Kalnoky M, Peck R, et al. A Recombinant Positive Control for Serology Diagnostic Tests Supporting Elimination of Onchocerca volvulus. PLoS Negl Trop Dis. 2016;10:e0004292
- Hernández-González A, Moya L, Perteguer MJ Herrador Z, Nguema R, Nguema J, et al. Evaluation of onchocerciasis seroprevalence in Bioko Island (Equatorial Guinea) after years of disease control programmes. Parasit Vectors. 2016;9:509..
- Herrador Z, Garcia B, Ncogo P, Perteguer MJ, Rubio JM, Rivas E, et al. Interruption of onchocerciasis transmission in Bioko Island: Accelerating the movement from control to elimination in Equatorial Guinea. PLoS Negl Trop Dis. 2018;12:e0006471..
