# Supplementary material for: Approaching onchocerciasis elimination in Equatorial Guinea: Near zero transmission and public health implication
Source: Infect Dis Poverty. 2024 Nov 14;13:86. doi: 10.1186/s40249-024-01254-9 (PMC11562331; doi:10.1186/s40249-024-01254-9)
Supplement: Supplementary file 14 — Additional file 14: Supplement Table 1. Participants enrolled by community and date of visit. [file 40249_2024_1254_MOESM14_ESM.docx]

**Supplement Table 1. Participants enrolled by community and date of visit.**

71 communities (6 more than the theoretical 65 initially selected) were visited to reach the established sample size previously designed: 3900 participants, 300 participants per district (sampling unit, n=13).

| **PROVINCE** | **DISTRIT** | **COMMUNITY** | **N** | **VISIT DATE** |
| --- | --- | --- | --- | --- |
| Litoral  (n=921) | Bata | Ndjiacom | 78 | 12/11/2019 |
|  |  | Nconvia | 24 | 15/11/2019 |
|  |  | Bicome | 84 | 15/11/2019 |
|  |  | Adjimelang | 28 | 12/11/2019 |
|  |  | Alum | 53 | 18/11/2019 |
|  |  | Nkoekien | 35 | 12/11/2019 |
|  |  | TOTAL | **302** |  |
|  | Mbini | Mbomo | 58 | 13/11/2019 |
|  |  | Bicucbini | 67 | 14/11/2019 |
|  |  | Engong | 59 | 14/11/2019 |
|  |  | Bolondo | 87 | 13/11/2019 |
|  |  | Abia | 34 | 18/11/2019 |
|  |  | Mitom | 15 | 18/11/2019 |
|  |  | TOTAL | **320** |  |
|  | Cogo | Ncoho | 50 | 14/11/2019 |
|  |  | Nzang Nnam | 53 | 15/11/2019 |
|  |  | Odjuma | 40 | 15/11/2019 |
|  |  | Mitong | 39 | 14/11/2019 |
|  |  | Vabe | 99 | 18/11/2019 |
|  |  | Meyang | 22 | 30/11/2019 |
|  |  | TOTAL | **303** |  |
| **C**entro Sur (n=909) | Niefang | Nzung Coro | 55 | 16/11/2019 |
|  |  | Bisun | 79 | 16/11/2019 |
|  |  | Bicaba | 81 | 16/11/2019 |
|  |  | Nvom | 93 | 16/11/2019 |
|  |  | TOTAL | **308** |  |
|  | Evinayong | Ayantangan | 60 | 20/11/2019 |
|  |  | Ebolowa | 75 | 20/11/2019 |
|  |  | Bikurga | 92 | 20/11/2019 |
|  |  | Abog Nsu | 75 | 20/11/2019 |
|  |  | TOTAL | **302** |  |
|  | Akurenam | Ayaesong | 59 | 21/11/2019 |
|  |  | Ngolonsork | 48 | 21/11/2019 |
|  |  | Akelayong Mbam | 93 | 21/11/2019 |
|  |  | Akurnam Esacora | 37 | 21/11/2019 |
|  |  | Ebian | 66 | 21/11/2019 |
|  |  | TOTAL | **303** |  |
| Wele-Nzas (n=1217) | Aconibe | Asok Abia | 32 | 22/11/2019 |
|  |  | Afanam | 94 | 22/11/2019 |
|  |  | Ebom | 57 | 22/11/2019 |
|  |  | Etom Asokne | 80 | 22/11/2019 |
|  |  | Bibas Ncodjuen | 27 | 22/11/2019 |
|  |  | Odjambouga | 14 | 22/11/2019 |
|  |  | TOTAL | **304** |  |
|  | Nsork | Ebomicú | 22 | 23/11/2019 |
|  |  | Nsork zona B | 42 | 25/11/2019 |
|  |  | Ngong Mocom | 33 | 23/11/2019 |
|  |  | Nvom | 66 | 23/11/2019 |
|  |  | Abama | 6 | 23/11/2019 |
|  |  | Alum Oyek | 48 | 23/11/2019 |
|  |  | Mocula | 56 | 23/11/2019 |
|  |  | Nzumu | 31 | 23/11/2019 |
|  |  | TOTAL | **304** |  |
|  | Mongomo | Ngolom | 27 | 25/11/2019 |
|  |  | Ebang | 105 | 25/11/2019 |
|  |  | Mebam Cdo | 91 | 25/11/2019 |
|  |  | Yenvam Melen | 60 | 25/11/2019 |
|  |  | Nmokien | 20 | 25/11/2019 |
|  |  | TOTAL | **303** |  |
|  | Añisok | Bikugu | 53 | 26/11/2019 |
|  |  | Odeng Cdo | 20 | 26/11/2019 |
|  |  | Añisok Yeken | 12 | 26/11/2019 |
|  |  | Mongola | 55 | 26/11/2019 |
|  |  | Acam Cdo | 78 | 26/11/2019 |
|  |  | Temelon | 82 | 26/11/2019 |
|  |  | TOTAL | **300** |  |
| Kie Ntem (n=902) | Nsoc Nsomo | Nfem-Mikó (Mengui) | 37 | 27/11/2019 |
|  |  | Mbarangun | 23 | 27/11/2019 |
|  |  | Ngumu | 69 | 27/11/2019 |
|  |  | Abiara Esatop | 25 | 27/11/2019 |
|  |  | Menga Eseng | 28 | 27/11/2019 |
|  |  | Oborunku | 35 | 27/11/2019 |
|  |  | Asok Sunga | 46 | 27/11/2019 |
|  |  | Eves | 38 | 28/11/2019 |
|  |  | TOTAL | **301** |  |
|  | Ebibeyin | Misana | 100 | 28/11/2019 |
|  |  | Ncoambe | 100 | 28/11/2019 |
|  |  | Bitet | 29 | 28/11/2019 |
|  |  | Ndumu | 71 | 28/11/2019 |
|  |  | TOTAL | **300** |  |
|  | Micomeseng | Mokom | 75 | 29/11/2019 |
|  |  | Beayop | 127 | 29/11/2019 |
|  |  | Mefem Ayop | 23 | 29/11/2019 |
|  |  | Nsang Oyek | 76 | 29/11/2019 |
|  |  | TOTAL | **301** |  |
| **TOTAL** |  | **71 COMMUNITIES** | **3951** |  |
