# Supplementary material for: Approaching onchocerciasis elimination in Equatorial Guinea: Near zero transmission and public health implication
Source: Infect Dis Poverty. 2024 Nov 14;13:86. doi: 10.1186/s40249-024-01254-9 (PMC11562331; doi:10.1186/s40249-024-01254-9)
Supplement: Supplementary file 15 — Additional file 15: Supplement Table 2. Onchocerciasis seroprevalence by district, mainland Equatorial Guinea from both visits. [file 40249_2024_1254_MOESM15_ESM.docx]

**Supplement Table 2. Onchocerciasis seroprevalence by district, mainland Equatorial Guinea from both visits.**

| **Province /**  **District** | **Population*** | **Tested** | ****Positive cases** | **Seroprevalence** | **95% *CI*** | ****UnD**  **cases** |  |
| --- | --- | --- | --- | --- | --- | --- | --- |
| LITORAL | | | | | | | |
| Bata | 309345 | 303 | 0 | 0 | 0.0–0.0 | 0 |  |
| Mbini | 28662 | 319 | 0 | 0 | 0.0–0.0 | 0 |  |
| Kogo | 29341 | 303 | 0 | 0 | 0.0–0.0 | 0 |  |
| CENTRO SUR | | | | | | | |
| Acurenam | 23614 | 303 | 0 | 0 | 0.0–0.0 | 0 |  |
| Evinayong | 56664 | 302 | 1 | 0.33 | 0.0–1.8 | 0 |  |
| Niefang | 61708 | 308 | 0 | 0 | 0.0–0.0 | 0 |  |
| WELE-NZAS | | | | | | | |
| Akonibe | 24346 | 304 | 0 | 0 | 0.0–0.0 | 0 |  |
| Mongomo | 88326 | 303 | 2 | 0.67 | 0.1–2.3 | 0 |  |
| Nsork | 16421 | 304 | 0 | 0 | 0.0–0.0 | 0 |  |
| KIENTEM | | | | | | | |
| Añisok | 62,24 | 300 | 1 | 0.33 | 0.0–1.8 | 1 |  |
| Ebebiyín | 94019 | 301 | 3 | 1 | 0.2–2.9 | 0 |  |
| Micomeseng | 51725 | 301 | 2 | 0.66 | 0.1–2.4 | 0 |  |
| Nsok-Nsomo | 37920 | 300 | 2 | 0.67 | 0.1–2.4 | 1 |  |
| **All** | **885015** | **3951** | **11** | **0.28** | **0.1-0.5** | **2** |  |

* Based on 2015 census.

** Number of positive or undetermined samples of anti-OV16 antibodies in ELISA considering first and second visits.

UnD: Undetermined
